# The Importance of Being Imperfect: Structure and Function of Bacterial Amyloid

**DOI:** 10.1002/advs.202517090

**Published:** 2025-12-05

**Authors:** Samuel Peña‐Díaz, Yanting Jiang, Zhefei Zhang, Anders Daugberg, Pedro Ferreira, Marcos López Hernández, Chandrika Mittal, Maria Joao Ramos, Jan Skov Pedersen, Morten Kam Dahl Dueholm, Cao Qin, Huabing Wang, Daniel E. Otzen

**Affiliations:** ^1^ Interdisciplinary Nanoscience Center (iNANO) Aarhus University Gustav Wieds Vej 14, DK Aarhus C 8000 Denmark; ^2^ Department of Clinical Laboratory, Key Laboratory of Clinical Laboratory Medicine of Guangxi Department of Education, Guangxi Key Laboratory of Enhanced Recovery after Surgery for Gastrointestinal Cancer the First Affiliated Hospital of Guangxi Medical University Shuangyong Road 6, Guangxi Zhuang Autonomous Region Nanning 530021 China; ^3^ Department of Chemistry and Bioscience Aalborg University Fredrik Bajers Vej 7H Aalborg Ø 9220 Denmark; ^4^ Bio‐X Institutes, Key Laboratory for the Genetics of Developmental and Neuropsychiatric Disorders Ministry of Education Shanghai Jiao Tong University Shanghai 200030 China; ^5^ Faculdade de Ciencias Universidad do Porto Porto 4169‐007 Portugal

**Keywords:** CsgA, FapC, functional amyloid, Greek key motif, solenoid

## Abstract

Amyloids, once viewed solely as pathological hallmarks, are now recognized as widespread and versatile functional protein assemblies. Bacterial functional amyloids (FuBAs), particularly curli (CsgA) from *Escherichia coli* and FapC from *Pseudomonas*, have emerged as paradigms for understanding amyloid structure, assembly, and function. The recent cryo‐EM‐based structure of FapC, together with others’ combined cryo‐EM and integrative computational studies on CsgA, reveal a β‐solenoid fold stabilized by imperfect repeats, producing fibrils of exceptional stability and low polymorphism, whose biogenesis is tightly controlled through dedicated accessory factors, ensuring precise secretion and nucleation. FuBAs not only scaffold biofilms but also display intrinsic catalytic activity, expanding the biochemical repertoire of extracellular matrices. They also exhibit hierarchical mechanical properties ranging from GPa stiffness at the fibril core to kPa elasticity in hydrated biofilms. FuBA operons are phylogenetically widespread, with repeat variation contributing to sequence diversity and functional adaptability. FuBAs might be seen as evolutionary intermediates between disordered peptides with significant self‐interaction tendencies and highly structured globular proteins. Their simple structures make them robust platforms for biomaterial engineering. Understanding the interplay between sequence repeats, fibril architecture, and emergent functions opens avenues for harnessing amyloids as programmable nanomaterials with applications in catalysis, synthetic biology, and biofilm control.

## Graphical abstract

Functional bacterial amyloids such as CsgA and FapC have been widely studied to understand the relationship between aggregation and function. The recently solved structure of FapC reveals a Greek‐key motif in which extensive hydrogen bonding and packing interactions formed by residues in conserved imperfect repeats stabilize a β‐solenoid core. FuBA fibrils display impressive mechanical and catalytic properties and are a very promising scaffold for the development of new biomaterials.

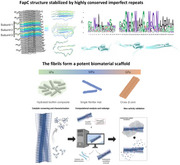

## 1 Amyloid: From Pathology to Functionality

Protein aggregation in the form of amyloid formation is commonly associated with devastating degenerative disorders like Parkinson's and Alzheimer's diseases. Pathology arises from the formation of toxic species, rather than loss‐of‐function of the native monomer. As the end state of these aggregation processes, the resulting amyloid shows a large structural diversity that does not exclusively rely on the sequence of the protein, but also on environmental conditions.^[^ ^1^ ^]^ Furthermore, their formation is preceded by a highly heterogeneous mixture of coexisting structures, including protofibrils and oligomers, varying in complexity and size. While the fibrils retain some intrinsic toxicity and play a key role in the spreading of the aggregates, the pre‐fibrillar constructs, particularly the oligomers, are considered the most toxic,^[^ ^2^ ^]^ with toxicity inversely proportional to size^[^ ^3^, ^4^, ^5^, ^6^ ^]^ but proportional to the extent of exposure of hydrophobic surfaces.^[^ ^7^, ^8^, ^9^, ^10^ ^]^

**Figure 1:**
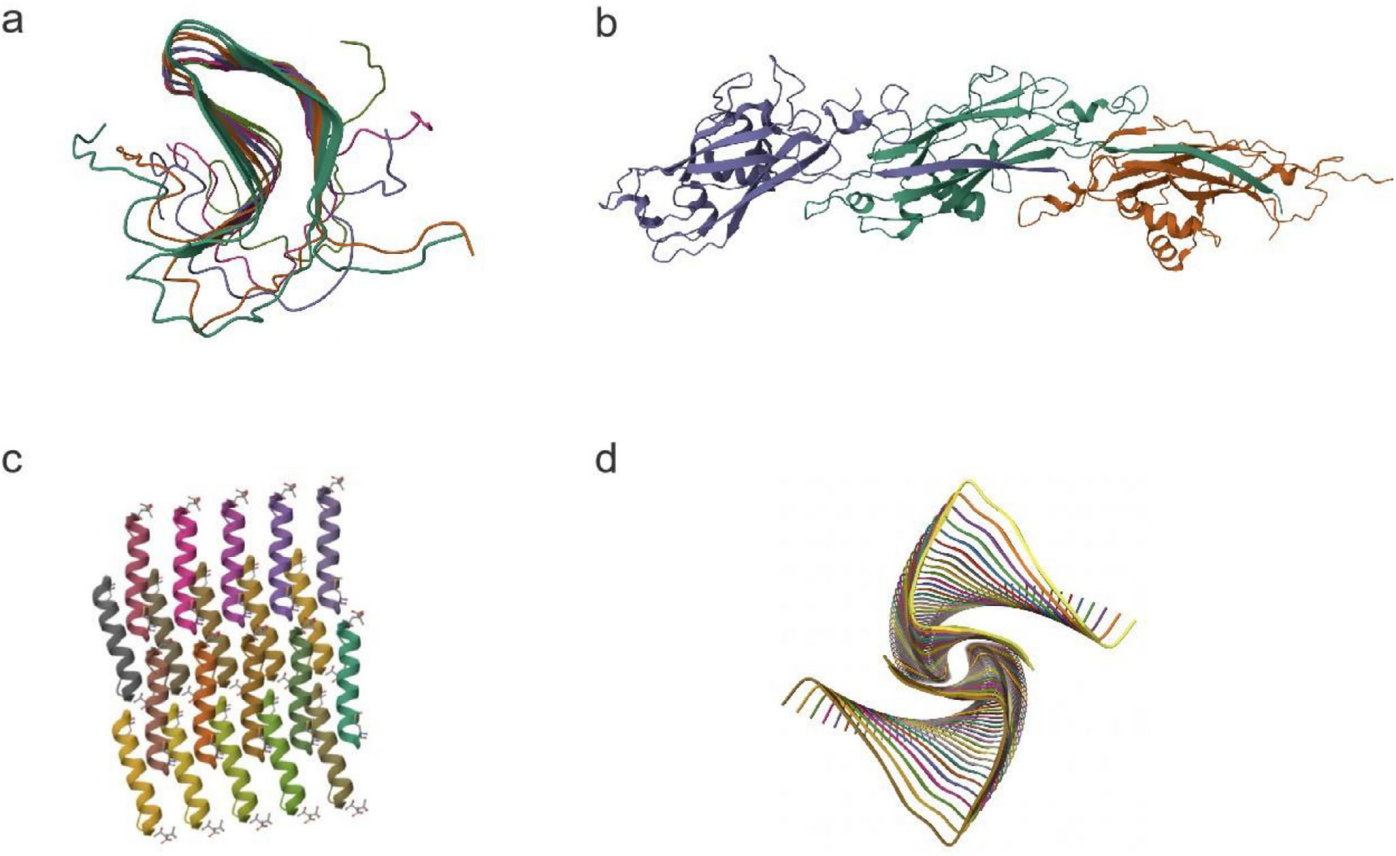
a) Structure of the HET‐s (218‐289) prion in its amyloid form obtained by solid‐state NMR (PDB: 2RNM). b) Cryo‐EM structure of a TasA scaffold (PDB: 8AUR). c) Crystal structure of the virulent PSMα3 peptide forming a cross‐α amyloid‐like fibril (PDB: 5I55). d) Structure of biofilm‐forming functional amyloid PSMα1 from *Staphylococcus aureus* (PDB: 9ATW).

However, pathological human amyloid (PaHa) is far from the only way in which amyloid manifests itself in our world. Amyloid, formed by many different types of proteins, plays essential roles in all kingdoms of life.^[^ ^11^, ^12^, ^13^ ^]^ In mammals, >30 different peptide and protein hormones are stored in secretory granules of the endocrine system as reversible amyloids;^[^ ^14^ ^]^ their lack of toxicity is attributed to a highly controlled and spatially constrained aggregation process, which only initiates once the pro‐hormone is processed in the secretory granules.^[^ ^14^ ^]^ Also, skin pigmentation in mammals is regulated by Pmel17 amyloid fibrils, which contribute to the appropriate processing of melanin precursors, avoiding the toxicity derived from melanin polymerization.^[^ ^15^ ^]^ Pmel17 undergoes a complex maturation process that culminates in the melanosomes, driven by the highly controlled proteolytic release of amyloidogenic fragments.^[^ ^16^ ^]^ Functional amyloids also play a role in epigenetic inheritance and memory.^[^ ^17^ ^]^ In fungi such as *Podospora anserina*, nontoxic HET‐s amyloid fibrils (**Figure** **1**a) are essential for the regulation of programmed cell death^[^ ^15^, ^18^, ^19^ ^]^ as follows. *P. anserina* can grow in two different and incompatible strains, i.e., HET‐s (with prion‐like properties) and HET‐S (without these properties). Upon fusion of two incompatible strains, the HET‐s amyloid fibrils induce a toxic conformational change of HET‐S, which initiates cell death. Other good examples in *Saccharomyces cerevisiae* include the prion‐like proteins Sup35 and Ure2p.^[^ ^20^, ^21^, ^22^ ^]^ In their soluble forms, Sup35 acts as a translation termination factor and Ure2p as a mediator of nitrogen catabolite repression.^[^ ^20^, ^21^, ^22^ ^]^ However, both proteins can form transmissible amyloid fibrils that inhibit the monomers’ function and lead to a new phenotype, facilitating cellular adaptation to a changing environment.^[^ ^20^, ^21^, ^22^ ^]^ The existence of amyloids in plants is still controversial, but has been suggested to contribute to protein storage and germination in seeds.^[^ ^23^ ^]^

## 2 Functional Bacterial Amyloid as the Main Source of Novel Amyloid Insights

Notwithstanding amyloids’ many different origins, bacteria provide the richest molecular and physiological insight. Functional bacterial amyloids (FuBAs) are involved in diverse functions, particularly as a major biofilm component. In *Bacillus subtilis*, the 233‐residue TasA, a natively disordered protein in the cytoplasm, was initially described as a functional bacterial amyloid due to its tinctorial properties.^[^ ^24^ ^]^ Nevertheless, its definition as a canonical functional amyloid remains controversial, as independent studies have demonstrated its capacity to form both amyloid and non‐amyloid fibrils.^[^ ^25^, ^26^, ^27^, ^28^ ^]^ Thus, TasA has been shown to form cross‐β structures as reported by X‐ray diffraction. Additionally, Fourier‐Transformed Infra‐Red, soluble NMR, and solid‐state NMR revealed polymorphic fibrils of TasA composed of cross‐β fibril core functionalized with α‐helixes.^[^ ^26^, ^29^ ^]^ However, another study reported TasA self‐assemblies unable to bind Congo Red or thioflavin T, as well as lacking the canonical X‐ray and CD amyloid fibril features,^[^ ^27^ ^]^ while a cryoEM study reported a scaffold in which Ig‐like domains self‐assemble by donor‐strand exchange (Figure 1b).^[^ ^28^ ^]^ In both cases, TasA aggregation is nucleated after its secretion by TapA, an accessory protein that also facilitates anchoring.^[^ ^30^ ^]^ Interestingly, TasA has been suggested to play a role in *B. subtilis* sporulation and to have antimicrobial properties, suggesting an alternative function of these amyloid fibrils.^[^ ^31^ ^]^ In *Staphylococcus aureus*, the phenol‐soluble modulins (PSMs) constitute a group of peptides found as soluble α‐helical monomers that display antimicrobial capacity, biofilm‐dismantling properties, and cytotoxic and pro‐inflammatory activities.^[^ ^32^ ^]^ Remarkably, PSMs have been shown to be capable of transitioning from the soluble α‐helical conformation to an amyloid fibril that contributes to biofilm scaffolding (PSMα1 and PSMα4) or displays antimicrobial properties (PSMα3). While PSMα1 and PSMα4 have been shown to form cross‐β rich amyloid fibrils, PSMα3 is unique in being able to form different polymorphs rich either in cross‐β or cross‐α structures,^[^ ^33^, ^34^, ^35^, ^36^ ^]^ depending on environmental conditions (Figure 1cd).

The most common functional bacterial amyloids (FuBAs) are CsgA and FapC, the major amyloid components of the curli system in *Escherichia coli* ^[^ ^37^ ^]^ and the Fap system in *Pseudomonas*, respectively.^[^ ^38^ ^]^ These two amyloid constructs are essential for biofilm scaffolding. CsgA and FapC have remarkable structural and functional properties. In their native hosts, they increase cell stiffness and hydrophobicity^[^ ^39^ ^]^ by protecting both the cell surface and the extracellular matrix with a physically and chemically very robust layer of β‐sheets.^[^ ^40^ ^]^ As programmable “smart” materials, they have been used for applications ranging from aquaplastic^[^ ^41^ ^]^ to regulated sequestration of toxic heavy metals^[^ ^42^ ^]^ and functionalization of the amyloid backbone for catalytic properties,^[^ ^43^ ^]^ all of which we have recently reviewed.^[^ ^44^ ^]^

While the aggregation propensity of both pathological and functional amyloids is imprinted into their sequence, they differ in the mechanisms that lead to their aggregation. Pathological amyloids are based on proteins that have evolved to remain soluble as monomers under normal conditions, and their aggregation correlates with abnormal conditions, including genetic mutations/duplications, altered pro‐protein processing, truncations, or dysregulated proteo‐ and homeostasis. In contrast, functional amyloid formation is highly controlled as detailed below. Despite these differences, the two groups of proteins can cross‐talk both constructively and destructively. Fibrils of CsgA produced in the gastrointestinal tract by endogenous *E. coli* can stimulate aggregation of α‐Syn in the brain^[^ ^45^ ^]^ and FapC accelerates aggregation of Aβ,^[^ ^46^ ^]^ while monomeric Aβ impedes and even reverses aggregation of both FapC and CsgA,^[^ ^47^ ^]^ as we have reviewed recently.^[^ ^44^ ^]^

Prompted by our recent presentation of the fully experimentally determined structure of FapC from *Pseudomonas* sp. UK4,^[^ ^48^ ^]^ we here describe the structural underpinnings of the many striking features of bacterial functional amyloid and the new insights this provides into the proteins’ formation and functionality.

## 3 The Cryo‐EM Structure of FapC Reveals the Importance of Imperfect Repeats

**Figure 2:**
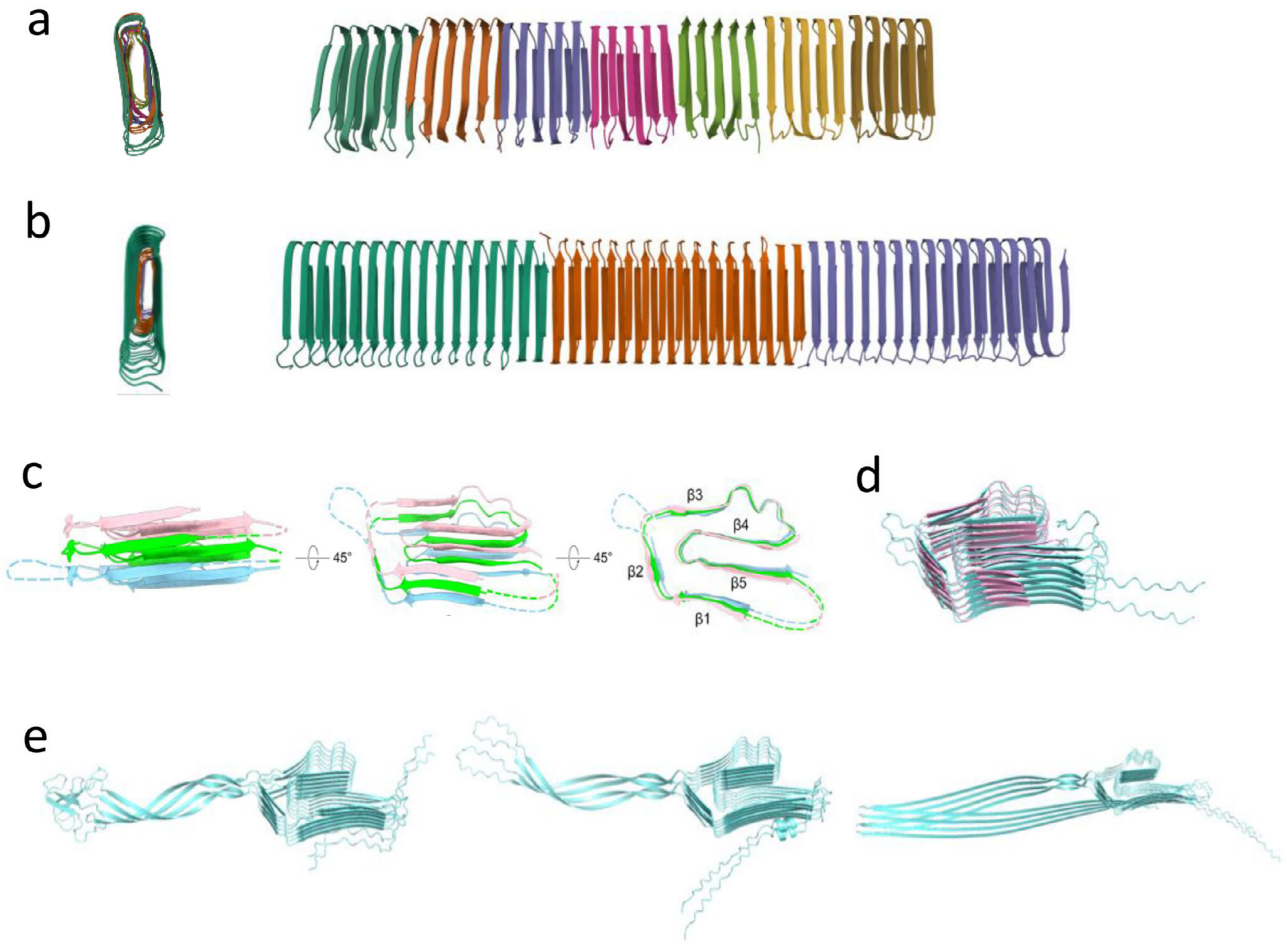
a) Top and side views of the Cryo‐EM of CsgA fibrils from *E. coli* (PDB: 8ENQ). b) Top and side views of the Cryo‐EM of R15.5 fibrils from *Pontibacter korlensis* (PDB: 8C50). c) The FapC UK4 structure of cryo‐EM analysis. d) Structural homology between AF3‐predicted (blue) and cryo‐EM‐described (pink) FapC structure. e) Predicted structures of FapC variants (PAO1, Pf5, and F1) based on AF3.

FuBA exhibits significant differences from pathological amyloid proteins, not only in its spatial conformation but also in its precisely controlled assembly process, which demonstrates unique structural programmability and environmental adaptability.^[^ ^49^ ^]^ In recent years, with the resolution revolution of cryo‐EM technology and the deep integration of computational prediction methods such as AlphaFold (AF), researchers have been able to systematically analyze the fibril architecture and assembly mechanism of FuBA at the near‐atomic level.^[^ ^48^, ^50^, ^51^ ^]^ Although recent advances in cryo‐EM and helical reconstruction have enabled the determination of several FuBA structures, detailed information at the side‐chain level remained scarce until recently. For CsgA, the lack of helical twisting of the fibrils limited cryo‐EM analysis to medium resolution and required cryoEM volumes to be combined with AlphaFold2 predictions for Remaut and coworkers to propose an atomic model for the curli protofibrils (**Figure** **2**b), revealing a typical β‐solenoid fold and a conserved cross‐β amyloid core.^[^ ^51^ ^]^ This fold was also observed in an independent cryo‐EM study by Bu *et al.* ^[^ ^50^ ^]^ However, due to an actual resolution of only 4–6 Å, the experimental density did not allow clear differentiation of side‐chain conformations (Figure 2a). Both studies fitted the predicted monomer model to the electron density map to construct the fibril model. However, details on CsgA connectivity and assembly mechanism into fibrils remain unclear due to the low resolution.^[^ ^52^, ^53^ ^]^

Recently, we were able to obtain a major breakthrough by using cryo‐EM on FapC from *Pseudomonas* strain UK4.^[^ ^48^ ^]^ Thanks to the twisting of the fibrils, we were able to determine the structure at 3.2 Å resolution, marking the first clear visualization of FapC at this level of detail. The sequence of FapC from UK4 consists of three imperfect repeats (IRs), each of which forms a Greek key‐shaped fibrillar unit that is stacked on top of each others within the monomer and interconnected by flexible linkers (Figure 2b). Importantly, the high‐resolution density map enabled unambiguous differentiation of side‐chain conformations among IR1‐IR3 and revealed, at the atomic level, a heterogeneous stacking pattern between layers within this FuBA fibril. As a result, FapC has become the first IR‐containing FuBA to be resolved to side‐chain precision. The covariation of IRs hydrophobic residues forms a globular‐protein‐like hydrophobic core in which residues in adjacent IRs are sterically matched. This is a significant difference compared to PaHa, which usually lacks IRs or is made from short peptides.

The high‐resolution structure of FapC enables clear visualization of interface details between monomers, revealing FapC connectivity and assembly mechanism. FapC and CsgA are quite similar in terms of structural characteristics since they are both FuBAs with imperfect repeats. They both have highly conserved Asn and Gln residues, which engage in an extensive side chain hydrogen‐bonding network to stabilize both the β‐arch motifs fold and β‐sheet. The difference is that CsgA forms a simple β‐arch while FapC forms a more complicated Greek key arch with both hydrophobic and hydrophilic core and long loop between imperfect repeats, suggesting a greater level of evolution towards a globular protein fold. The structure of FapC not only fills a critical gap in high‐resolution structural knowledge of functional bacterial amyloids but also provides a robust structural basis for developing precise interventional strategies against amyloid‐mediated biofilm formation and for designing novel biomaterials.

## 4 High Consistency Between the Cryo‐EM Structure of FapC and AF3 Predictions Despite Differences in β‐Chain Planarity

Systematic comparison between the experimentally determined cryo‐EM structure and the AF3 predicted model is essential for validating structural accuracy and elucidating the underlying folding mechanisms. In the FapC UK4 structure, the overall AF3 prediction aligns closely with the experimental electron density map, particularly in the spatial arrangement of conserved residues—such as Asn53/118/181 and Gln89/150/227—that stabilize the Greek key shape through an extensive hydrogen‐bond network.^[^ ^48^ ^]^ However, while AF3 predicts that β‐strands within the same repeat unit lie in the same plane, the experimental structure reveals distinct out‐of‐plane tilting and a staggered arrangement^[^ ^48^ ^]^ (Figure 2c). This observed non‐planar conformation facilitates the formation of additional hydrogen bonds between adjacent repeating units and optimizes sidechain packing within the hydrophobic core, thereby significantly enhancing the fibril's overall rigidity and stability.^[^ ^48^ ^]^ The structurally conserved regions of the FapC variants PAO1, Pf5, and F1 predicted by AF3 are consistent with this conformation, though the intervening linker regions are predicted to have some rather unusual folds that require further structural analysis (Figure 2d). FapC PAO1 and FapC Pf5 predicted structures, previously reported in ref. [48], exhibited a pLDDT value of ≥70% and pTM/ipTM scores of 0.59/0.54 and 0.69/0.65, respectively, indicating a relatively high‐quality prediction. In case of FapC F1, linker regions complicate the prediction of the fibrillar structure and therefore reduce its accuracy as indicated by the pLDDT value (70–90% for the fibril core and 50–70% for the linker regions) and the pTM/ipTM scores (0.4/0.33). AF3's predictive capability provides strong support for the reliability of the amyloid core shown in the cryo‐EM model and greatly minimizes potential misinterpretations arising from reconstruction algorithms or data inaccuracies. Nevertheless, high‐resolution experimental data remain indispensable for correcting and refining subtle yet critical conformational variations, such as β‐strand displacement and torsion.

## 5 Differences Between FuBA and PaHA: Sequence Composition and Structural Polymorphism

Although both FuBA and PaHA share a fibrous cross‐β sheet architecture, they exhibit systematic differences that reflect distinct evolutionary origins and functional constraints. This is also reflected in their degree of structural polymorphism. FuBA fibrils are generally long, straight, and unbranched, with highly uniform morphology and architecture.^[^ ^48^, ^50^, ^51^, ^54^ ^]^ The IRs found in FapC promote conformational consistency through optimized stacking and hydrogen‐bond networks, thereby suppressing structural variations,^[^ ^48^ ^]^ and this will likely also be revealed for CsgA once a high‐resolution structure is available. PaHA exhibits pronounced polymorphism, frequently forming multiple fibrillar subtypes with distinct morphologies under varying conditions. This structural heterogeneity is closely associated with variations in disease manifestation and progression.^[^ ^54^ ^]^ FuBA proteins typically feature regularly repeating imperfect motifs enriched in polar residues such as Asn and Gln, which facilitate extensive hydrogen bonding. Such imperfect repeats are extremely common in FuBAs, though varying between different proteins in architecture, number, composition, and conservation, and evolutionarily selected to efficiently modulate their aggregation.^[^ ^55^ ^]^ The hydrophobic residues also display periodic patterns that support cooperative folding and fibril elongation.^[^ ^54^ ^]^ In contrast, the aggregation core of PaHA is often driven by short, hydrophobic segments. Their sequence composition is largely determined by the native protein's amino acid profile rather than by evolutionary optimization for ordered self‐assembly.^[^ ^56^ ^]^ Linear aggregation predictors usually predict hotspots in key aggregation regions more efficiently in PaHAs than FuBAs (see below). However, the efficiency of predicting FuBAs aggregation hotspots varies from predictor to predictor, with a tendency to predict only terminal IRs, which in any case are more amyloidogenic than the sequences in between, due to their role in intermolecular contacts between monomers. This not only complicates the determination of a consensus region but also the selection of the most appropriate predictor and interpretation of the outcomes. Therefore, from a computational perspective, combining these linear predictions with evolutionary conservation analysis could be a smart albeit preliminary strategy to shed light on the sequential determinants of FuBAs aggregation.

Aggrescan,^[^ ^57^ ^]^ Amylpred,^[^ ^58^ ^]^ Waltz^[^ ^59^ ^]^ or CamSol^[^ ^60^, ^61^ ^]^ perfectly predict Aβ42's fibril core and α‐Syn's NAC‐region as well as regions P1, P2, which are responsible for the oligomer‐to‐fibril transition. However, results for FuBA are mixed. For CsgA, the predictors mostly identify IR1 (Aggrescan, Waltz and CamSol) and IR5 (Aggrescan, Amylpred and CamSol); only CamSol predicts the internal repeats as aggregation hotspots but with significantly lower propensity, consistent with the observed higher aggregation propensity of IR1 and IR5, and the modulator role of IR2 and IR4.^[^ ^62^, ^63^, ^64^ ^]^ As for FapC, only Aggrescan and CamSol can predict IR2 and IR3 as aggregational hotspots, while IR1 is hardly observed with CamSol. Most predictors suggested aggregational hotspots located in the linker regions, confirming their experimentally observed amyloidogenicity.^[^ ^65^ ^]^ Altogether, these predictors are biased towards regions driving protein–protein interactions and thus fibril elongation, but show large variability in predicting FuBAs hotspots compared to PaHAs.

## 6 How the FuBA Structure is Formed: Biogenesis and Controlled Aggregation

**Figure 3:**
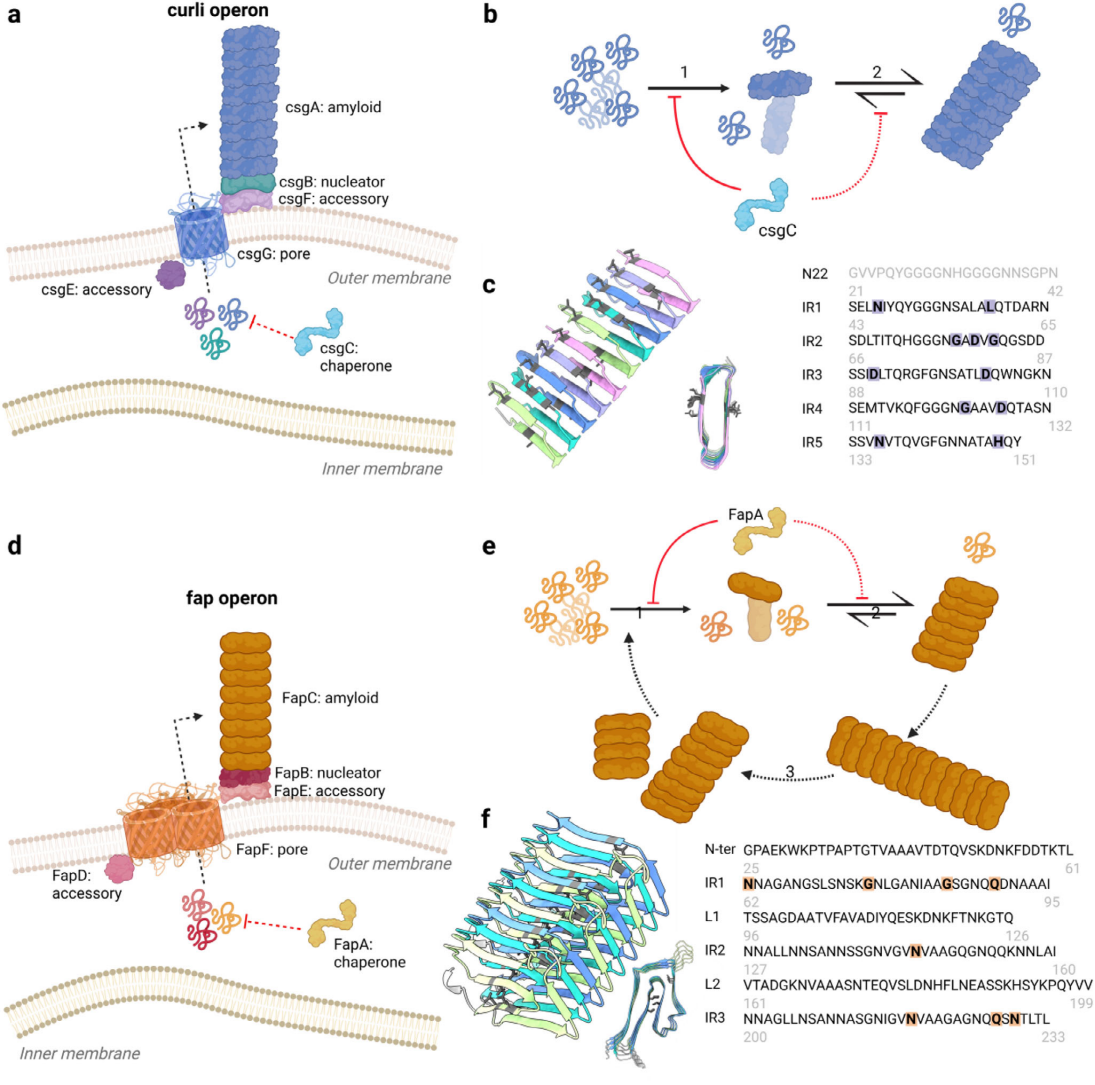
Schematic representation of the formation of functional bacterial amyloids. a) Representation of curli in vivo aggregation machinery. b) Mechanism of CsgA aggregation and inhibition in vitro, predominantely dominated by (1) primary nucleation and (2) elongation. c) Structural and sequential determinants of CsgA gatekeeper residues (gray and shaded residues). d) Representation of curli in vivo aggregation machinery. e) Mechanism of FapC aggregation and inhibition in vitro, predominantly dominated by primary nucleation (1) and elongation (2), but also contributed by fragmentation processes (3). f) Structural and sequential determinants of FapC gatekeeper residues (gray and shaded residues).

FapC and CsgA have followed independent evolutionary trajectories that converged in similar aggregation machinery, involving a periplasmatic chaperone, a transport system, and a nucleator factor.^[^ ^13^ ^]^ The overall result is a highly controlled process that includes compartmentalization, lower exposure of hydrophobic patches, higher structural organization and compaction, a well‐defined pre‐processing of the protein to release the amyloidogenic segment, and a set of complementary proteins that guarantee the solubility of the major amyloid component prior to fibril formation. However, their end structures are different, and there are also differences in the details of biogenesis. CsgA curli fibrils are produced in a complex and highly conserved regulatory system (**Figure** **3**a) that encompasses multiple *csg* genes in two different operons (*csgBAC* and *csgDEFG*),^[^ ^66^, ^67^ ^]^ whose expression is mediated by the transcription regulator CsgD.^[^ ^66^, ^67^ ^]^ CsgA from *E. coli* consists mainly of five ≈20‐residue highly conserved, IRs with amyloid‐prone properties.^[^ ^66^, ^67^ ^]^ *En route* to the cell exterior, it avoids periplasmatic aggregation due to the periplasmic chaperone CsgC,^[^ ^66^, ^67^ ^]^ and subsequently exits via the outer membrane‐associated curli components CsgG and CsgF, together with the periplasmatic CsgE.^[^ ^66^, ^67^ ^]^ Recent CryoEM atomic‐level resolution of the CsgG‐CsgF secretion complex shows that CsgG formed a β‐sheet transmembrane barrel with the C‐terminal region facing the periplasmatic region, which interacts with the N22 segment of CsgA prior to its secretion.^[^ ^68^, ^69^ ^]^ CsgF forms an α‐helical structure that is translocated to the cellular surface and interacts via N‐terminus with the CsgG channel and anchors the CsgA‐nucleator agent, CsgB, through its C‐terminal region.^[^ ^67^ ^]^ CsgE remains in the periplasmatic region, where it interacts with both CsgG C‐terminal helixes and with the IRs of CsgA, facilitating translocation and preventing its aggregation.^[^ ^67^ ^]^ The exported CsgA is integrated into the growing end of the CsgA fibril, which extends from the bacterial cell surface through contact with CsgB, which also contains IRs. Consistent with this, both CsgA and CsgB aggregate rapidly in vitro with positive cross‐seeding activities.^[^ ^67^ ^]^ Aggregation of CsgA is dominated by primary nucleation processes and fibril elongation,^[^ ^64^ ^]^ i.e., formation of the first nuclei and their elongation are limiting (Figure 3b). Direct in situ AFM observations further confirmed this mechanism by showing that CsgA undergoes rapid, one‐step nucleation without detectable oligomeric intermediates, and then elongates in a polar manner with characteristic stop‐and‐go kinetics.^[^ ^70^ ^]^ IR1 and IR5 alone can form amyloid‐like structures^[^ ^62^, ^63^, ^64^ ^]^ while so‐called gatekeeper residues in IR2‐4 (typically Asp/Glu) reduce amyloidogenic propensity of internal repeats^[^ ^62^ ^]^ acting as an intrinsic safeguard against premature aggregation during secretion, but not the stability of the ensuing fibrils,^[^ ^67^ ^]^ avoiding premature aggregation (Figure 3c). Within IR1 and IR5, mutations of surface‐exposed residues, which are expected to participate in the monomer–monomer interaction, also modulate the amyloidogenic propensity of the repeat.^[^ ^62^ ^]^ CsgA aggregates to the same fibril state over a wide range of environmental conditions^[^ ^71^ ^]^ (even in the presence of denaturing agents as urea^[^ ^72^ ^]^) highlighting the robustness and evolutionary optimization of its functional self‐assembly. Charge plays a modest role in fibrillation,^[^ ^67^ ^]^ again reflecting the robustness of the self‐assembly process. Altogether, these findings demonstrate that CsgA biogenesis integrates multiple layers of quality control—including chaperoning, compartmentalization, regulated secretion and nucleator‐guided templating—to ensure efficient yet tightly controlled formation of curli amyloid fibers at the cell surface.

Despite this highly evolved propensity for aggregation, many different chaperones (not just its natural inhibitor CsgC) block CsgA polymerization, preferentially targeting primary nucleation^[^ ^73^ ^]^ but also elongation. These chaperones are more efficient towards CsgA than FapC, suggesting either a sequence‐ or structure‐dependent anti‐aggregational mechanism or alternative aggregational processes.^[^ ^73^ ^]^ Nonetheless, the two proteins aggregate through broadly similar mechanisms, primarily relying on primary nucleation and elongation.^[^ ^74^ ^]^ This contrasts with PaHa, which are more prone to secondary processes such as fragmentation and lateral nucleation processes, leading to explosive and thus less‐controlled growth.

Fap and curli similarities also extend to the genetic systems for producing them. Six different *fap* genes are organized in a single operon (*fapABCDEF*) (Figure 3d) with FapC as the major fibril subunit. Analogous to CsgA, periplasmic aggregation of FapC (which is natively unfolded as monomer) is prevented by FapA, an intrinsically disordered chaperone^[^ ^75^ ^]^), and secretion is mediated by an outer membrane pore made of FapF,^[^ ^76^ ^]^ a trimeric β‐barrel whose opening is regulated by an α‐helical segment connected to a coiled‐coil.^[^ ^76^, ^77^ ^]^ The secretion, however, also depends on FapD, a cysteine protease that is thought to process the N‐terminus of FapE and the periplasmatic region of FapF as an essential step for FapC and FapB secretion.^[^ ^76^, ^78^ ^]^ Once secreted, the fap fibril is mainly constituted by FapC, but its formation is nucleated by FapB and the fibril is anchored to the cell membrane via FapE,^[^ ^78^, ^79^ ^]^ analogous to CsgB and CsgF, respectively.

In vitro both FapC or FapB form amyloid structures with cross‐seeding capacities.^[^ ^79^ ^]^ The aggregation of FapC is usually fitted by a fragmentation‐dominated process; however, primary nucleation and elongation still play a major role.^[^ ^48^, ^80^, ^81^ ^]^ (Figure 3e). This stronger dependence on secondary processes compared to CsgA may be linked to differences in fibril structure, with the Greek‐key‐like motif of FapC resembling the pathological amyloid architecture.^[^ ^48^ ^]^ Interestingly, the deletion of one or more of the repeats increases the fragmentation propensity,^[^ ^80^, ^82^ ^]^ which suggests that increasing the number of repeats is stabilizing, although the linker region itself is also able to form fibrils.^[^ ^80^ ^]^ At the level of individual residues (Figure 3f), mutations in the core of the fibril have different effects: Asn62, Gly84, and Asn218 are essential for fibril formation, while mutations of Gly75, Gln89, Asn145, Gln227, and Asn229 substantially modify the formation process but not fibril stability.^[^ ^48^ ^]^ Further studies are needed to decipher the role of other regions in the FapC fibrillation process.

Like CsgA, FapC aggregates over a wide range of conditions,^[^ ^79^, ^83^ ^]^ and this is modulated by a multitude of chaperones, peptides, and small molecules^[^ ^73^, ^84^, ^85^, ^86^ ^]^ that mainly affect the primary nucleation and elongation processes. The natural inhibitor FapA prevents in vitro aggregation of FapC and FapB by targeting primary nucleation and elongation, respectively.^[^ ^75^ ^]^ Computational analysis using AlphaFold suggested that FapA's affinity for FapC decreases as the fibril is formed.^[^ ^49^ ^]^

## 7 Bioinformatic Analysis of the Fap and Curli Operons: An Update

**Figure 4:**
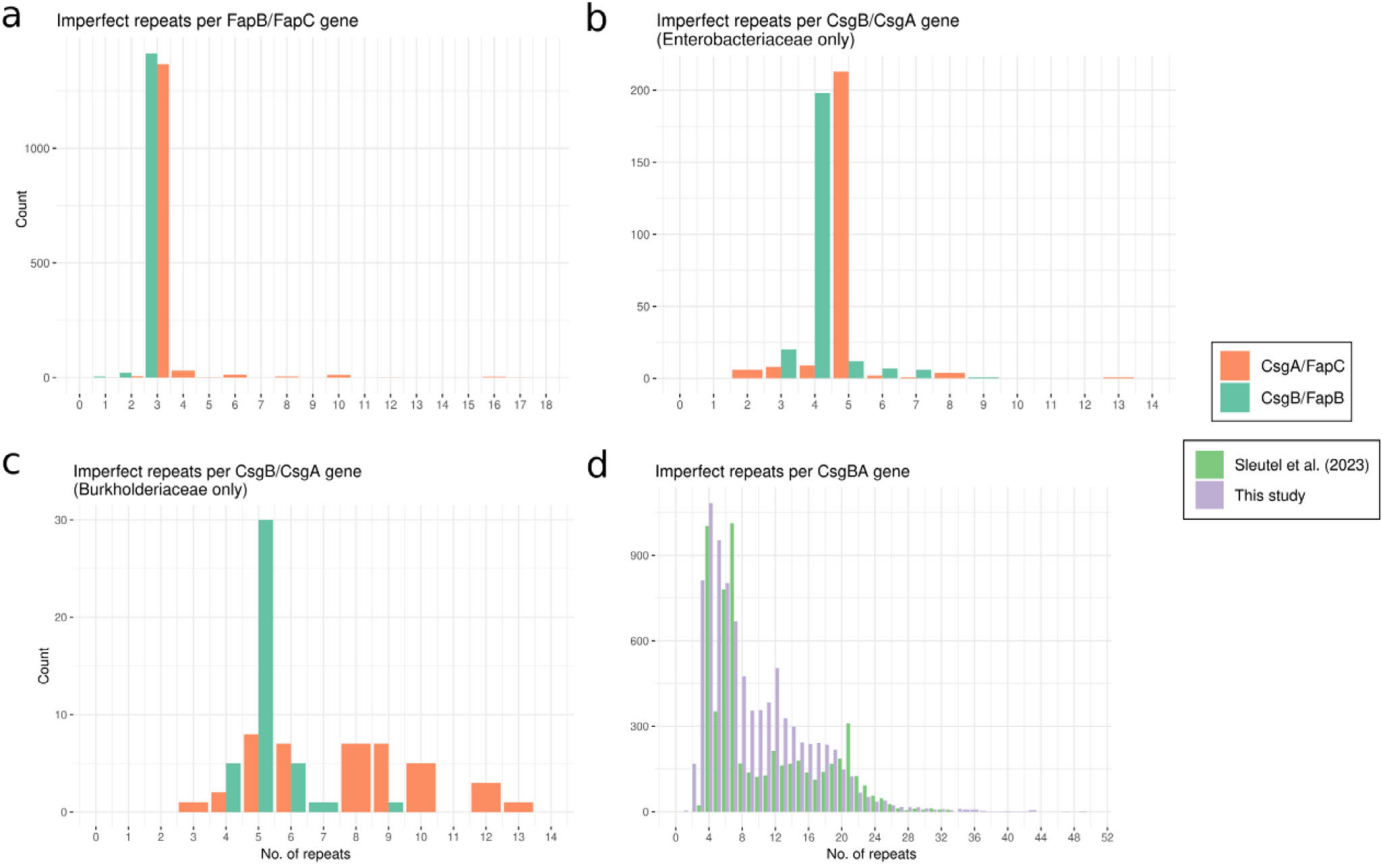
Histograms depicting the number of imperfect repeats in subunit (CsgA/FapC) and nucleator (CsgB/FapB) proteins in a) *fap* operons, b) Enterobacterial Curli operons, and c) Burkholderial Curli operons. d) Number of imperfect repeats in CsgB/A homologs detected by Sleutel *et al*.^[^ ^51^ ^]^ (data from the article) versus the number of imperfect repeats in CsgB/A homologs detected in this study. All code for our analysis is found at https://github.com/AOHD/amyloid_review.

**Figure 5:**
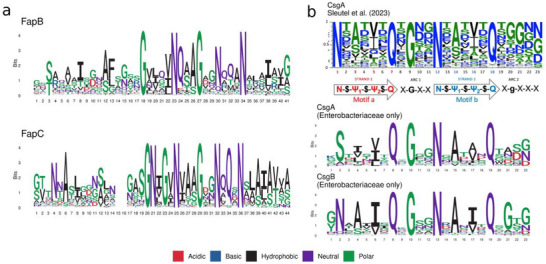
Sequence conservation across imperfect repeats. a) Sequence logos of the most commonly occurring residues in FapB and FapC. b) Sequence logos of the most commonly occurring residues in CsgA from Sleutel *et al.* (reproduced under terms of the CC‐BY license.^[^ ^51^ ^]^ Copyright 2023, The Authors, published by Springer Nature), as well as sequence logos of the most commonly occurring residues in Enterobacterial CsgA and CsgB (this study).

As evolutionarily optimized amyloid‐formers, the sequences of FapC and CsgA provide unique insight into how to design robust amyloids. Indeed, the operons encoding the proteins for both Fap and Curli production are phylogenetically widespread.^[^ ^87^, ^88^ ^]^ In our study on the structure of FapC earlier this year,^[^ ^48^ ^]^ we screened all species‐representative genomes in the 214.1 release of the Genome Taxonomy Database (GTDB)^[^ ^89^ ^]^ for homologs to the FapB/C IR sequence as well as the other genes in the *fap* operon. When we required the FapB/C IR homologs to be within 5000 bp of other Fap homologs, ≈95% of the resulting gene clusters either contained all 6 *fap* genes, representing the canonical *fapABCDEF* operon, or were missing a FapA homologue (resulting in a *fapBCDEF* operon). This analysis, which used the Hidden Markov Models (HMMs) constructed in ref. [90], represented the most comprehensive screening for *fap* operons across the bacterial tree of life to date and showed for the first time that the presence of exactly three IRs in FapC and FapB is almost universal. We have now updated this analysis using the 2025 release of GTDB (v226), which contains ≈40% more species representatives than the 214.1 release from 2023 (85,205 in v214.1, 143,614 in v226). When requiring homologs of the FapB/C IRs to be within 5000 bp of another *fap* gene homolog, > 90% of the resulting 1602 gene clusters displayed the canonical *fapABCDEF/fapBCDEF* operon synteny. These operons allowed us to specify the FapB/C homologs as either FapB or FapC based on their position in the operon, which in turn allowed us to investigate the differences in the number of repeats between these two genes. Additional methodological details are available in the Supporting Information (SI) file. The results reaffirmed that both FapB and FapC almost invariably contain three IRs (**Figure** **4**a) and led to the creation of updated sequence logos for both genes, which closely resembled those from our previous study (Figure 5a).

How does this compare with curli? To address this, we performed a similar screening as described above, using HMMs for the CsgB/A IRs and CsgC,D,E,F,G, and H, which were constructed in ref. [87]. When requiring homologs of the CsgB/A IRs to be within 5000 bp of another curli gene homolog, a much higher variance was seen in the compositions of the detected curli operons compared to the *fap* operons. The five most abundant gene cluster compositions (3846/4723 detected gene clusters, ≈81%) encoded for CsgA/B+CsgEFG, CsgA/B+CsgDEFG, CsgA/B+CsgH, CsgA/B+CsgFG, and CsgA/B+CsgCDEFG. These gene compositions and their corresponding gene cluster syntenies were also seen when we performed the first HMM‐based curli screening on a much smaller refseq database^[^ ^87^ ^]^ (10 000 bacterial genomes^[^ ^91^ ^]^). In the present study, due to the much higher diversity in operon structure in the Curli operons, we only designated CsgA/B homologs as either CsgA or CsgB in curli operons with a complete *csgBAC* operon (317/4723, ≈9% of detected gene clusters). Most of these operons (245/317, ≈77%) were found in the Enterobacteriaceae family. The enterobacterial CsgB and CsgA almost invariably contained 4 and 5 IRs, respectively (Figure 4b). In Burkholderiaceae, which contained the next‐highest number of *csgBAC* operons, most CsgB homologs contained five repeats, while CsgA contained a seemingly random number of repeats between three and fourteen (Figure 4c). Recently, Remaut and co‐workers^[^ ^51^ ^]^ showed that the number of repeats in CsgB/A homologs in general (without differentiating between CsgB and CsgA) spans from four to 62, with local maxima at 5–8 and 15–22 repeats. Our analysis broadly agrees with the maximum and minimum number of repeats (3‐52), but the distribution is markedly different, with local maxima shifted to 3–8 and 9–14 repeats (Figure 4d). Since the same HMMs were used in both analyses, the discrepancy between the repeat distributions is most likely due to the different genome databases used. The Remaut group used the RefSeq bacterial genome database, which differs from GTDB by rarely allowing the inclusion of genomes from uncultured bacteria, such as those from metagenomes. Consequently, RefSeq contains significantly fewer species‐representative genomes (at the time of writing, there are 21.3k reference genomes in the RefSeq database), many of which belong to the most well‐studied and easily culturable taxa. While our results generally align well with the pioneering work from the Remaut group, we argue that the histogram obtained from our GTDB‐centered analysis is more representative of the repeat distribution amongst Curli‐producing bacteria.

Due to the large difference in repeat numbers between families (Figure 4bc), family‐specific sequence logos were made for Enterobacterial CsgA and CsgB (**Figure** **5**b). The sequence logos bear a large resemblance to the sequence logo created by the Remaut group,^[^ ^51^ ^]^ which contained two N‐X‐Ψ_1_‐X‐Ψ_2_‐X‐Q motifs (motifs a and b) where Ψ_n_ indicates hydrophobic residues and X indicates no conservation between repeats. These motifs are bridged by an X‐G‐X‐X sequence. In enterobacterial CsgA, the leading asparagine residue is swapped for a serine. This was known to be the case for *E. coli* already, but here we show that this substitution is conserved at the family level. In CsgA, motif b is followed by an X‐X‐X‐(n/g/d) sequence, while in CsgB the sequence following motif b is X‐g‐X‐(n/g/d), with small letters indicating incomplete conservation of the amino acid. Both sequences are one residue smaller than the one from the Remaut group: X‐X‐X‐(n/g/d/s). The differences between the CsgA logo from Remaut and the ones in this study can be explained by the different approaches to finding the repeat sequences to construct them from. In our case, we focused on the enterobacterial CsgA and CsgB proteins involved in the CsgBAC operon. The Remaut group used regex to capture the canonical QX_10_Q repeat motif (and permutations of it). The sequence logo from Remaut might represent the global sequence of the CsgA repeat motif, while the logos in our present work likely reflect the specific enterobacterial CsgA and CsgB motifs.

**Figure 6:**
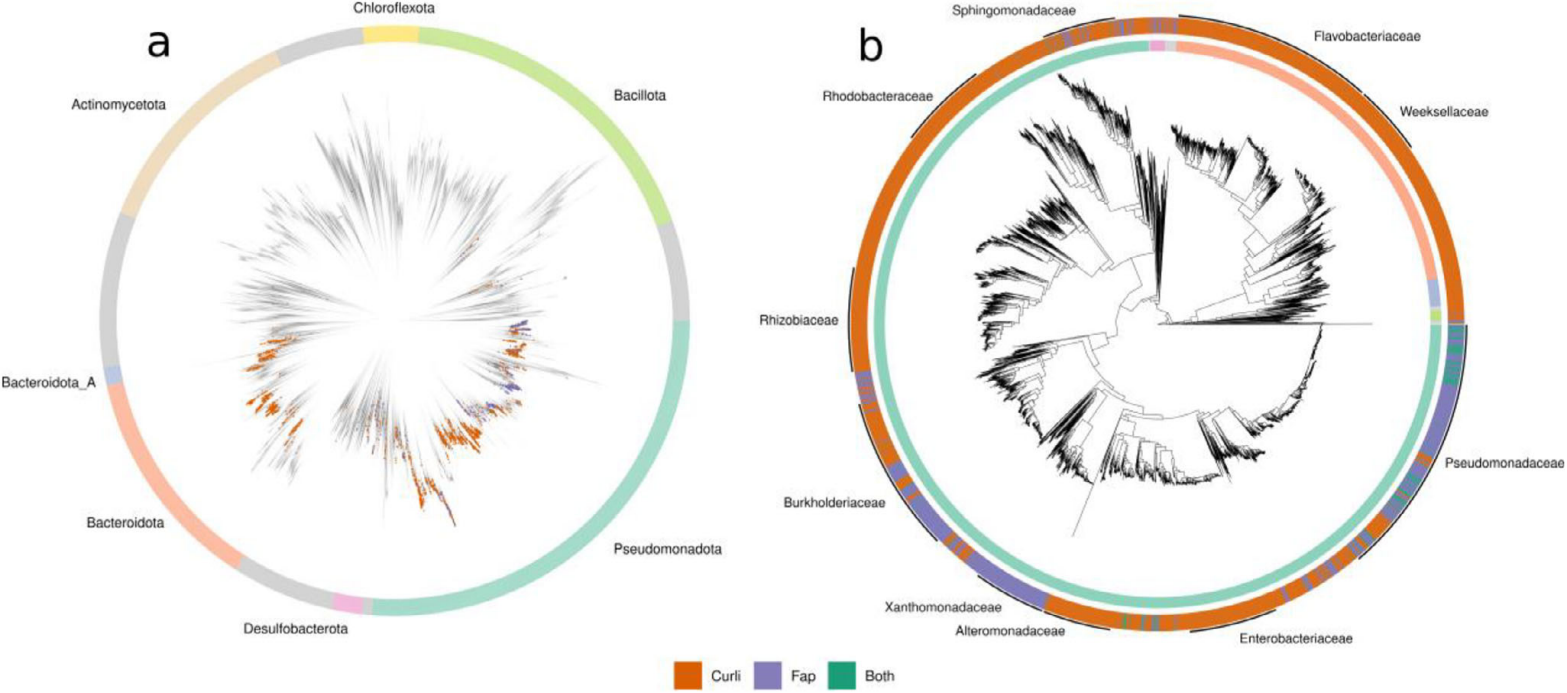
Phylogenomic analysis of FuBA. a) Phylogenomic tree of species representatives from GTDB v226. Dots on the tree indicate genomes where either curli operons, Fap operons, or both have been detected. The heatmap around the tree shows the major phyla in the tree (Remaining phyla = gray). b) Phylogenomic tree of just the GTDB species representative genomes in which a Fap operon, a curli operon, or both have been detected (n = 5579). The inner heatmap shows the major phyla in the tree (same color‐scheme as tree on the right), while the outer heatmap indicates the amyloid operon(s) detected in the genomes.

We detected curli and/or Fap operons in ≈4.2% (5779/136 646) of the species representatives in GTDB v226. The detected curli operons were much more widespread phylogenetically than the Fap operons. Both systems were found across many families in the Pseudomonadota phylum, as well as other phyla such as Desulfobacterota, Nitrospirota, and even Bacillota (**Figure** **6**ab). The presence of both operons in a genome seems to be genus‐specific, with *Pseudomonas_E* and *Shewanella* representing the most cases. Curli operons were also widespread in Bacteroidota and Bacteroidota_A, whereas no Fap operons were found in the genomes of these phyla.

In summary, while Fap operons are highly conserved in both gene synteny and repeat number—almost invariably maintaining three imperfect repeats in FapB and FapC—curli operons exhibit striking diversity in both operon composition and repeat architecture. This contrast suggests that Fap represents an evolutionarily “fixed” amyloid system optimized for robustness and stability, whereas curli appears to be a more flexible scaffold whose repeat number and operon structure vary across bacterial lineages. Together, these findings underscore two distinct evolutionary strategies for functional amyloid formation: one emphasizing conservation and reproducibility (FapC), the other adaptability and diversification across ecological niches (CsgA).

## 8 Low but Measurable Catalytic Properties of FuBA Provide an Experimental and Computational Platform for Functionalization

Amyloid proteins, particularly functional amyloids, have been widely studied as potential biomaterials due to their exceptional properties.^[^ ^44^ ^]^ A novel approach is to exploit or engineer catalytic properties,^[^ ^44^, ^92^, ^93^, ^94^, ^95^ ^]^ but the analysis of FUBA's intrinsic catalytic properties remains sparsely studied. Several PaHAs have recently been shown to perform hydrolytic reactions^[^ ^96^, ^97^, ^98^ ^]^ while the functional amyloid PSM from *Staphylococcus aureus* (PSMs) degrades β‐lactam antibiotics.^[^ ^99^ ^]^ Recently, we demonstrated that FapC fibrils from *Pseudomonas* sp UK4 can carry out esterase and lipase reactions in solution or functionalizing surfaces, even after repeated denaturing cycles (heat, chemical denaturants etc.).^[^ ^48^ ^]^ CsgA fibrils from *E. coli* K12 can also degrade ester and lipidic bonds (S. P.‐D. and D.E.O., unpublished results). Interestingly, the major fibril units (CsgA and FapC) are the least conserved components of their corresponding operons,^[^ ^90^ ^]^ suggesting that the fibril sequence could be evolutionarily modified to adapt the bacterial biofilm to the changing environment. Consistent with this, FuBAs such as curli fibers, but no other biofilm components, have recently been shown to play an active role in the protection against other predatory bacterium and viral infections.^[^ ^100^, ^101^ ^]^ These studies demonstrated that curli fibers built a physical barrier that selectively excluded predatory bacteria, phage dispersion, and cell adhesion. Intrinsic catalytic and protective properties could be an evolutionary selection of fibril properties that potentially enhance bacterial fitness, which immediately suggests the possibility of modifying CsgA and FapC fibrils to perform or enhance different catalytic reactions. This will require a further understanding of the mechanism underlying these intrinsic properties by combining computational tools with the recently solved structures of CsgA and FapC fibrils.^[^ ^48^, ^50^, ^51^ ^]^

We have already embarked on this process, using primarily molecular docking and classical molecular dynamics (cMD) simulations to elucidate substrate‐amyloid interactions and identify potential catalytic sites responsible for the intrinsic catalytic activity exhibited by functional amyloids. We have described the general approach in a methodological paper^[^ ^43^ ^]^; the rest of this section summarizes additional computational work which underscores our experimental observations and which is currently being prepared for publication. Recent studies have demonstrated the usefulness of these approaches in understanding amyloid catalysis at the atomic level. For instance, cMD simulations of PSMα3 amyloids revealed that specific Lys clusters are involved in the experimentally observed hydrolysis of nitrocefin.^[^ ^99^ ^]^ More computationally heavy quantum mechanics/molecular mechanics calculations provided quantitative insights into the energetics of amyloid catalysis, calculating activation barriers for the acylation step of 4‐Nitrophenyl Acetate (pNPA) hydrolysis on native glucagon amyloid fibrils and demonstrating a significant barrier reduction of 3.2 kcal mol^−1^ compared to the equivalent reaction in water.^[^ ^102^ ^]^ Within the field of catalytic amyloid, *p‐*Nitrophenyl derivatives, including 4‐Nitrophenyl Acetate (pNPA) and 4‐Nitrophenyl butyrate (pNPB), have become key tools to study potential catalytic behavior on the fibrils’ surfaces.^[^ ^48^, ^96^, ^97^, ^102^ ^]^ Their simplicity, availability, and ready degradability make them easy to implement when screening for potential hydrolytic processes. Similarly, their simple structure facilitates the computational analysis and redesign of the fibril catalytic moieties by reducing the computational time that more complex substrates could require, while also holding the potential for screening during the structural determination using cryo‐EM.

Building on these computational examples, we have conducted a comprehensive screen to identify potential catalytic sites on the available structures of CsgA and FapC using pNPA, pNPB, ampicillin, and nitrocefin as probe substrates. To eliminate potential bias in substrate placement and ensure thorough sampling of the amyloid surface, we employed i) blind molecular docking followed by six independent 500 ns cMD simulations for each substrate‐amyloid complex, and ii) six additional 500 ns cMD simulations with substrates placed randomly in the simulation box, allowing unbiased substrate‐amyloid encounters. Additional methodological details are available in the SI file.

Unlike biological enzymes that possess well‐defined catalytic pockets typically buried within the protein core, the catalytic sites on amyloid fibrils such as CsgA and FapC are expected to be surface‐exposed and flat and show low substrate specificity. To take this into account, we quantified the probability of occurrence of potentially reactive microenvironments along the fibril surface, rather than looking for catalytic sites from a lock‐and‐key perspective. We defined potential catalytic microenvironments based on three essential geometric and chemical criteria: 1) Oxyanion stabilization: the presence of at least one hydrogen bond donor within 3.2 Å of the carbonyl oxygen of the scissile bond (ester carbonyl for pNPA and pNPB; β‐lactam carbonyl for ampicillin and nitrocefin), which would stabilize the oxyanion intermediate formed during nucleophilic attack; 2) Nucleophile proximity: the presence of a nucleophilic group within 4 Å of the electrophilic carbon atom of the scissile bond, enabling direct nucleophilic attack; and 3) Water accessibility: the presence of water molecules within a 5 Å radius of the scissile bond.

**Figure 7:**
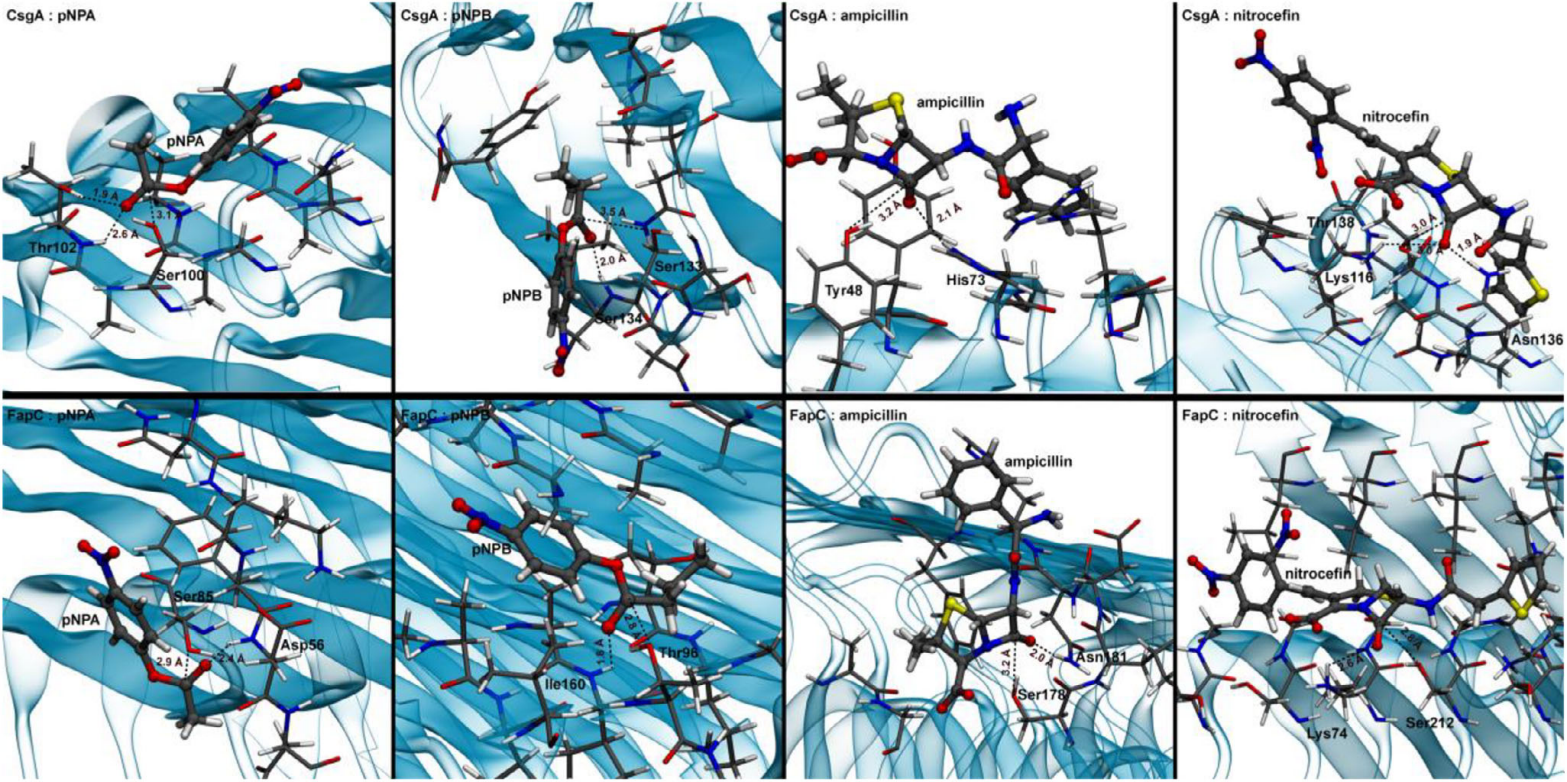
3D representations of the most frequent catalytic sites on CsgA (top) and FapC (bottom) identified in the cMD simulations for each amyloid:substrate pair (different columns). Each snapshot corresponds to the frame in which the nucleophilic residue is closest to the electrophilic carbon atom of the substrate throughout the trajectories. Substrates are shown as ball‐and‐stick, while all amyloid residues within 4.5 Å are depicted as sticks. Key distances, including nucleophile‐substrate and hydrogen bonds between the substrate's carbonyl oxygen and amyloid residues, are explicitly highlighted. The overall amyloid structure is illustrated in a light blue cartoon with a semi‐transparent surface.

Notably, despite the initial unbiased positioning of the substrates and unrestrained cMD simulations, catalytic conformations, simultaneously meeting the three criteria described above, were found in all amyloid:substrate pairs, highlighting the potential of native CsgA and FapC to hydrolyze the four substrates tested. Neither CsgA nor FapC exhibited well‐defined binding sites found in evolved enzymes. Rather, both systems display multiple weak binding sites and nucleophilic residues that interact transiently with substrates throughout the simulations. **Table** **1** summarizes the main results and the primary binding sites identified for each substrate‐amyloid combination, revealing that most substrates bind to distinct sites, each characterized by different geometric arrangements and chemical environments (**Figure** **7**).

**Table 1:** Summary of key metrics from molecular docking and classical molecular dynamics simulations for each amyloid‐substrate pair. Each value reported represents the average across twelve independent 500 ns cMD trajectories, totaling 6 µs per system. Six simulations were initiated from blind docking poses and six with substrates randomly placed in the simulation box, to ensure unbiased, thorough sampling of possible binding modes. The percentage of bound conformations indicates the fraction of total simulation time in which the carbonyl oxygen of the substrate's scissile bond forms at least one hydrogen bond with amyloid residues, using a donor–acceptor distance cutoff of 3.2 Å. The percentage of catalytic conformations refers to the fraction of simulation time meeting all of the following criteria simultaneously: (i) the substrate carbonyl oxygen is stabilized by the amyloid (≤3.2 Å); (ii) a nucleophilic group is positioned within 4 Å of the carbon of the scissile bond; and (iii) at least one water molecule is present within 5 Å of the scissile bond. The potential nucleophiles column lists the specific amyloid nucleophilic residues most frequently found within the catalytic conformations.

Substrate^a)^	Amyloid	% of cMD with bound conformations	% of cMD with catalytic conformations	Potential nucleophiles
Ampicilin	CsgA	14.4	0.02	Tyr48 Ser131
	FapC	38.0	4.23	Ser178 Ser189
Nitrocefin	CsgA	15.8	0.05	Thr61 Thr138
	FapC	30.2	2.22	Ser71 Ser212
pNPA	CsgA	10.4	0.54	Ser100 Thr102
	FapC	8.0	0.39	Ser85 Ser212
pNPB	CsgA	9.2	0.33	Ser133 Thr147
	FapC	14.6	0.26	Thr39 Thr96

^a)^ All production simulations were performed at 310.15 K and 1 bar using GROMACS 2025.3, employing the v‐rescale thermostat and C‐rescale barostat. Protein residues were described with the ff19SB force field, and water molecules were represented with the OPC3 model. Substrate parameters were generated using the Antechamber modules of AmberTools24, applying the GAFF2 atom types and RESP charges derived from HF/6‑31G(d) electrostatic potentials.

While our criteria aimed to identify regions with catalytic potential, the kinetic efficiency of each site will depend on the chemical and electrostatic nature of the local environment and the precise spatial arrangement of catalytic residues. The apparent catalytic efficiency ranking derived from binding analysis provides valuable insights into relative substrate preferences, but quantitative prediction of reaction rates will require quantum‐level calculations to accurately model transition state energetics and activation barriers for each identified catalytic microenvironment.

Future computational studies should prioritize the development of larger‐scale fibril models that incorporate multiple fibril assemblies to comprehensively capture the spectrum of intra‐ and inter‐fibril interactions. The catalytic sites identified in current studies, based predominantly on short models, may represent only a subset of the reactive environments present in mature amyloid fibrils. Inter‐fibril interfaces can create unique binding pockets and microenvironments that are absent in simplified models, potentially harboring catalytic sites with enhanced substrate binding and/or reaction kinetics. The computational modeling of such complex, large‐scale fibrillar systems presents substantial technical challenges, requiring advanced sampling methods and significant computational resources. Multiscale modeling strategies that combine atomistic detail with coarse‐grained representations of extended fibril structures may offer a viable path forward. However, the success of these endeavors will critically depend on close integration with high‐resolution experimental structures and in vitro studies to ensure that the computational models accurately reflect the structural complexity and dynamic behavior of native amyloid assemblies.

## 9 SAXS Provides Complementary Information on Folded and Dynamic Regions, Aided by AlphaFold

In contrast to our success with FapC UK4, we have not yet obtained high‐resolution structures of FapC fibrils from other species due to their lack of twisting in the fibril structure; such twisting provides repeating symmetry, which is necessary for computational reconstruction.^[^ ^103^ ^]^ For systems such as these FapC fibrils, where the absence of high‐resolution structural data constrains interpretation, small‐angle X‐ray scattering (SAXS) provides a complementary approach by delivering low‐resolution but solution‐relevant information.^[^ ^104^ ^]^ At low protein concentration, SAXS measures the form factor, *P*(*q*), which reflects the size and shape of the particle as a function of the modulus of the scattering vector, *q*. The form factor of the particles can be studied by two different approaches. In the first step, analytical expressions that describe the scattering of geometric shapes are fitted to the data, and in the second step, the theoretical scattering of high‐resolution structures, which account for the coordinates of each atom in the structure, is compared with the data. In line with the first approach, analytical models based on cylindrical form factors with elliptical cross‐sections have been widely applied to amyloid‐like fibrils.^[^ ^65^, ^105^ ^]^ These models often incorporate polymer‐like brushes to approximate flexible regions extending beyond the ordered fibril core, as the fibril length typically exceeds the experimental *q* range, and most of the measurable signal derives from the core of the cross‐sectional profile.

**Figure 8:**
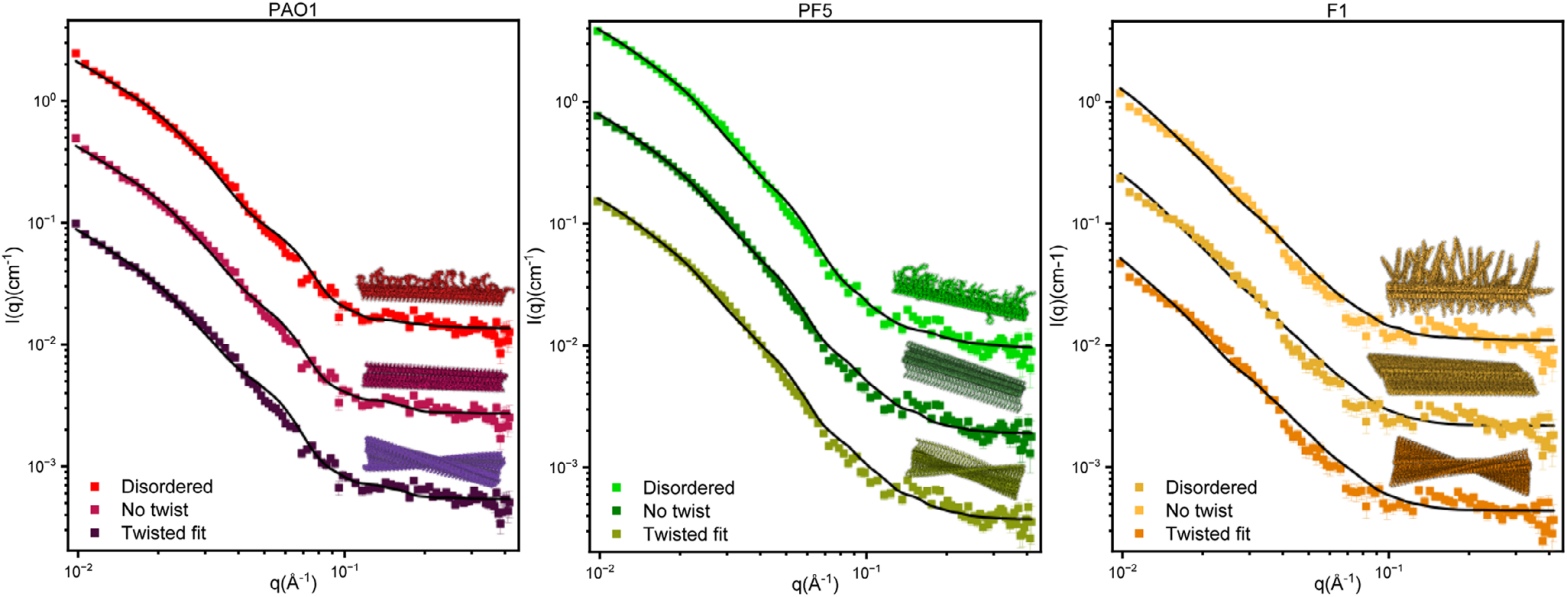
Experimental data modeling for three different FapC variants. Stacked fittings of the same experimental curves are shown for each variant. All models for each fibril were built using generative AI (reference copilot + ChatGPT) from monomers extracted from AlphaFold‐predicted small fibrils to avoid steric clashes during the translation of different copies and further rotated with a specific twisted angle (bottom curves). For the non‐twisted models, the angle was set to zero, while the specific domains of the side regions were allowed to rotate randomly for the disordered models. All resulting models were evaluated against the scattering data using AUSAXS.^[^ ^96^, ^97^, ^98^ ^]^ χ^2^ of each fit : a) PAO twisted: 6.1. PAO non‐twisted: 5.3. PAO disordered: 4.4. b) PF5 twisted: 8.6. PF5 non‐twisted: 7.1. PF5 disordered: 6.9. c) F1 twisted: 4.77. F1 non‐twisted: 4.7. F1 disordered: 4.1.

The second approach has become possible since recent developments in cryo‐electron microscopy (cryo‐EM) and AlphaFold‐based predictions now permit the construction of all‐atom fibril models, extending beyond simplified analytical representations and resulting in more detailed information. This provides a high‐resolution structural hypothesis that can be tested for consistency with the SAXS data. We previously demonstrated both approaches for different FapC variants,^[^ ^48^ ^]^ where excellent agreement was observed between scattering data, recorded in‐house^[^ ^106^ ^]^ for all variants, both for analytical and atomistic models of UK4, the simplest of the structures. Here, we elaborate upon the analysis and apply it to strains PAO1, PF5, and F1 (Figure 8), which contain more extensive flexible regions outside the fibril core. Note that due to the limited *q*‐range of the experiment, the overall length of the fibrils is not resolved, and the measured intensity originates mainly from the cross‐sectional structure of the fibrils.

The different fibril structures of all variants were built as follows. First, a monomer copy from AF3 predicted protofibrils was extracted and used as the starting structure to avoid clashes. Then, the fibril structures were obtained by translation and rotation of copied monomers using an AI‐generated script with CoPilot and ChatGPT. A second step was used to evaluate the introduction of disorder; selected regions of each monomer were allowed to rotate randomly, followed by an energy minimization step to reduce steric clashes and stabilize, resulting in five different models for each “disordered” fibril. Then, the agreement of the theoretical and the experimental scattering was evaluated using AUSAXS,^[^ ^107^ ^]^ which allows the calculation of the theoretical scattering of these massive structures using the Debye equation. Additional methodological details are available in the SI file. We then assessed the impact that the twist angle of the fibril core and the disorder in the flexible regions have on the scattering modelling.

Two important points emerge from our analysis: *1) The importance of twists*. Gratifyingly, fibril models generated from AlphaFold‐predicted monomers showed good agreement with SAXS data. Further refinement was achieved by accounting for the absence of helical twist along the fibril axis, which confounds structural determination by cryo‐EM as described above. These results emphasize the importance of incorporating subtle geometrical parameters into scattering models. *2) The problem of disorder*. Structural characterization of side chains and loop regions remains a central difficulty, as these features are highly dynamic and typically unresolved in AFM or other high‐resolution methods. Attempts to model such disorder by random loop rotations across monomers may improve the quality of fits (as shown in **Figure** **8**), but the introduction of disorder did not consistently improve the agreement of all models studied. Lack of improvement may be due to different factors, including the oversimplification of the disorder representation, partial rigidity within apparently flexible regions, and the increase in hydration associated with disordered segments. Another point is that the interference pattern is formed by each particle with itself, and for non‐disordered models, the flexible regions are placed on the average positions, meaning that the interference term between these regions and the core is still quite well represented. Furthermore, the flexible regions are a smaller fraction of the total structure.

The findings suggest some limitations of this approach in describing flexible regions, which still require complementary modelling from an analytical model. This remains a source of uncertainty when reconciling atomistic models with experimental scattering data. Although the use of atomic resolution models represents major progress in this area, further progress is likely to arise from integrative strategies combining more accurate structural predictions and molecular dynamics simulations with SAXS analysis, enabling conformational ensembles to be captured more accurately. Complementary information may be obtained from limited proteolysis, which can be used to selectively remove (and identify) dynamic regions and study the properties of the remaining core,^[^ ^108^ ^]^ an approach long established for PaHa but not yet applied to FuBA.

## 10 Material Properties of FapC and CsgA Resolved by AFM: A Scale‐Aware Perspective

Atomic force microscopy (AFM) has been instrumental in linking the molecular architecture of bacterial functional amyloids to their mechanical roles across scales relevant for both material science and microbiology. Reconciling AFM studies of *E. coli* curli (CsgA) and *Pseudomonas* FapC requires careful attention to what is measured (intrinsic fibril core, fibril mat, or hydrated biofilm), how it is measured (sharp‐tip force spectroscopy versus colloidal‐probe indentation), and the sample state (dry/adsorbed versus hydrated/composite).^[^ ^109^ ^]^ When these factors are considered, apparently discordant stiffness values form a consistent, hierarchical picture.

For curli (CsgA), AFM force–distance indentation of fibrillar mats produced by engineered *E. coli* typically yields mesoscale Young's moduli in the low tens of MPa. Rational sequence or compositional modifications, such as the addition of CsgB or linker engineering, shift these mesoscale moduli predictably, demonstrating tunable network mechanics under hydrated, mat‐like conditions.^[^ ^110^ ^]^ Single‐fiber or single‐molecule AFM studies report higher intrinsic stiffness estimates (tens to a few hundred MPa when diameter, persistence length, and bending are explicitly considered), reflecting direct sampling of the fibril backbone under axial or bending loads rather than through‐thickness indentation of mats.^[^ ^111^ ^]^ Together, these studies place curli assemblies in an MPa‐to‐GPa regime for biologically relevant, hydrated constructs, while higher intrinsic values emerge under constrained or dehydrated conditions.

FapC requires a similar scale‐aware interpretation. Colloidal‐probe AFM indentation of intact, hydrated *Pseudomonas* biofilms shows that Fap amyloid production increases bulk biofilm stiffness roughly twentyfold—from ≈0.1 kPa in Δfap strains to ≈2.0 kPa under overexpression—while also enhancing surface hydrophobicity and intercellular cohesion. These measurements report the effective modulus of a hydrated, heterogeneous composite (cells + extracellular polymeric substances + amyloid network) and therefore lie in the kPa range relevant to colony mechanics.^[^ ^39^ ^]^

By contrast, high‐resolution structural and AFM studies of FuBA FapC from *Pseudomonas* sp. UK4 directly probes the cross‐β protofilament core and reports GPa‐range stiffness for the highly ordered β‐sheet core.^[^ ^48^ ^]^ This difference reflects the measurement scale rather than contradiction: tightly packed, crystalline β‐sheet cores exhibit GPa‐scale Young's moduli due to dense hydrogen‐bonding and side‐chain packing. Importantly, the FuBA study identifies stacking of IRs as a stabilizing design principle. Repeat stacking produces a highly ordered, hydrogen‐bonded core that is mechanically stiff at the fibril backbone scale, even while permitting sequence imperfection and heterogeneous packing at larger scales.

Accurate interpretation requires information about sample hydration, AFM mode, tip radius, indentation depth, contact model, and loading geometry. Recent multimodal mechanical studies have begun to quantitatively resolve how these parameters translate into distinct elastic regimes across bacterial biofilms. For *E. coli* microcolony biofilms, Siri et al. combined oscillatory shear rheology and micro indentation to directly compare bulk and local mechanical responses of heterogeneous extracellular composites.^[^ ^112^ ^]^ Bulk rheology of homogenized biofilms revealed storage moduli (*G*′) in the range of 16–50 kPa, depending on extracellular matrix (ECM) composition, while local indentation of intact colonies showed Young's moduli between 30 kPa and 100 kPa. Biofilms containing both curli and phosphoethanolamine‐modified cellulose (pEtN‐cellulose) exhibited the highest compressive stiffness (≈97 kPa), whereas curli‐deficient or cellulose‐only biofilms were markedly softer (≈40 kPa). These results underscore that matrix composition and mesoscale architecture jointly determine the emergent elasticity of hydrated, heterogeneous biofilm materials.

At smaller scales, nanoscale AFM mapping of engineered *E. coli* curli mats has revealed intrinsic fibril stiffness values in the GPa range, with single‐fiber Young's moduli of 2–10 GPa, which can be further enhanced above 30 GPa through integration with carbon nanotube scaffolds.^[^ ^113^ ^]^ These high moduli correspond to direct measurements of dehydrated β‐sheet cores probed by sharp tips and are consistent with the dense hydrogen‐bonding and sidechain packing that are characteristic of amyloid fibrils. When placed in the broader context of Pseudomonas FapC systems, colloidal‐probe AFM indentation of intact, hydrated biofilms yields effective moduli ≈1–2 kPa, whereas nanoscale indentation of isolated FapC fibrils reveals GPa‐range stiffness for the ordered cross‐β cores.^[^ ^39^, ^48^ ^]^

**Figure 9:**
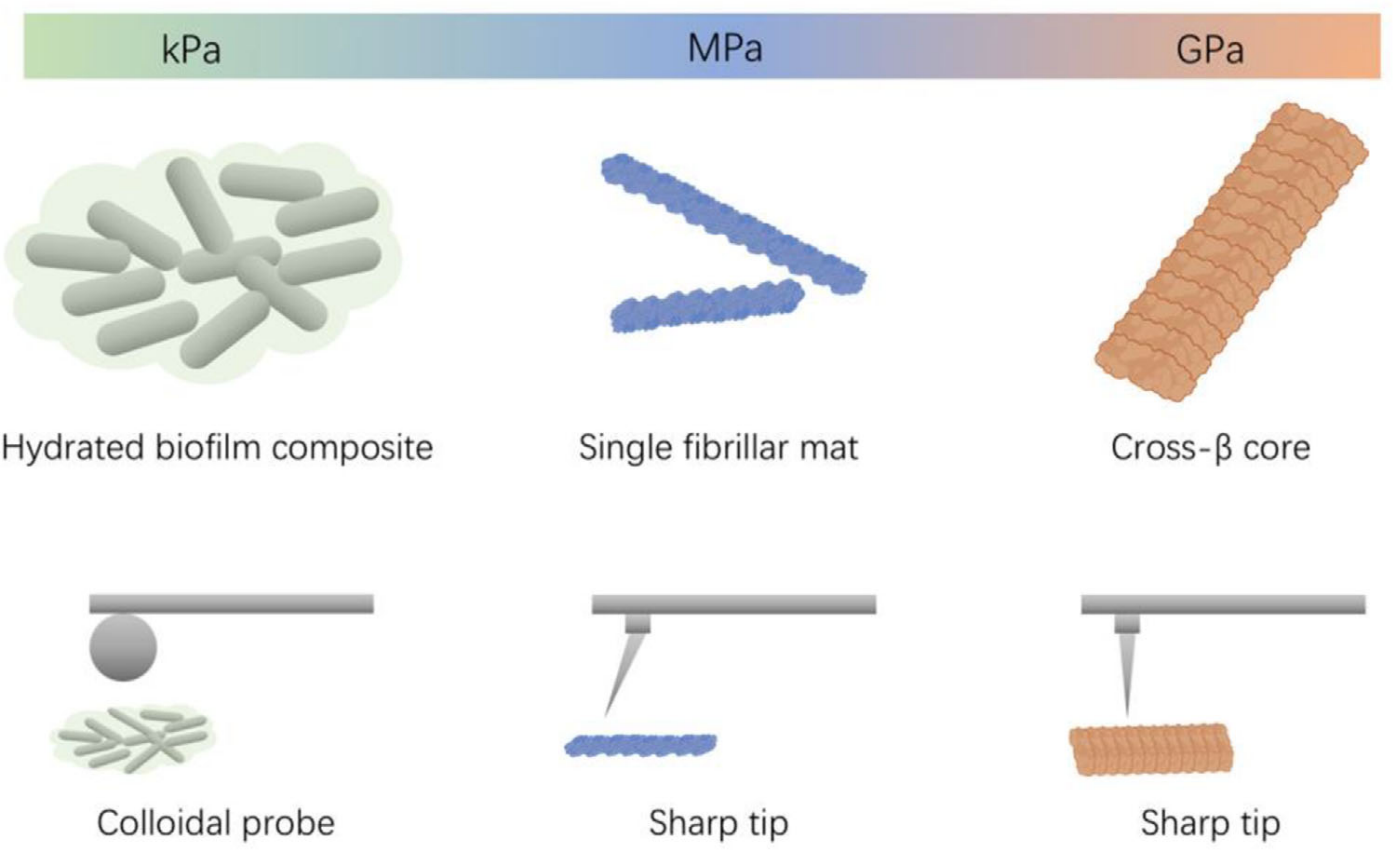
Hierarchical stiffness of bacterial amyloids resolved by AFM. Schematic overview of stiffness values measured for CsgA and FapC across different structural and biological scales. kPa regime: colloidal‐probe AFM indentation of intact, hydrated biofilms, where amyloids act within a heterogeneous composite of cells and extracellular matrix. MPa regime: AFM nanoindentation or bending of single fibers and fibrillar mats under hydrated conditions. GPa regime: sharp‐tip AFM probing of the highly ordered cross‐β core of individual fibrils (intrinsic backbone stiffness).

Together, these data establish a coherent, scale‐aware hierarchy of bacterial amyloid mechanics (**Figure** **9**):

– kPa: effective elasticity of hydrated biofilm composites probed by colloidal indentation or rheology;

– MPa: mesoscale stiffness of hydrated fibrillar mats and bundles under constrained loading;

– GPa: intrinsic stiffness of ordered, partially dehydrated β‐sheet cores probed by sharp‐tip AFM.

Such a quantitative framework reconciles previously disparate measurements and highlights how amyloid molecular architecture and matrix composition modulate mechanical behavior across scales relevant to microbiology and materials science. Integrating bulk rheology, mesoscale indentation, and nanoscale elasticity mapping will therefore be essential to fully link amyloid sequence design to emergent biofilm mechanics.

## 11 Beyond Simple Fibrils: Future Perspectives for FuBAs

In the course of the last decade, our view on FuBAs has matured from seeing them as peculiar biofilm components to acknowledging them as central model systems for amyloid biology. The emergence of high‐resolution structures of bacterial amyloids has transformed our understanding of how these fibrils achieve stability and function. In particular, the cryo‐EM structure of FapC and medium‐resolution models of CsgA reveal how IRs organize into β‐solenoid folds that enforce order and suppress polymorphism. This contrasts sharply with pathological human amyloids (PaHAs), where structural diversity underlies disease heterogeneity. Thus, FuBAs illustrate how evolution has solved the problem of robust fibril assembly, ensuring reproducibility rather than variability.

At the heart of this robustness lies the **modularity of IRs**. Repeats act as stabilizing units, yet their number and sequence can readily diversify, tuning fibril fragmentation, mechanical resilience, and aggregation kinetics. This dual role—as structural stabilizers and evolutionary “building bricks”—makes it tempting to speculate that FuBAs represent evolutionary intermediates between disordered peptides and globular proteins, harking back to prebiotic times where amyloidogenic (and therefore self‐assembling) peptides may have contributed to the origin of life by their templating and thus self‐replicating properties.^[^ ^114^ ^]^ In this light, FuBAs might be viewed as “living molecular fossils,” where the benefits of low polymorphism and high stability have allowed core elements of their sequence to be retained up to the present day. Their modular design not only suggests a simple mechanism for protein evolution but also provides a rational basis for engineering fibril properties in synthetic biology.

Beyond structural stability, FuBAs exhibit **functional diversification**. Experimental and computational analyses show that cavities in FapC and surface‐exposed nucleophiles create a promiscuous catalytic microenvironment.^[^ ^42^, ^82^, ^83^ ^]^ Although catalytic activity is weak compared to enzymes, it may still confer ecological benefits such as nutrient acquisition or antibiotic resistance, as observed for PSM amyloids degrading β‐lactams.^[^ ^99^ ^]^ These latent activities suggest that FuBAs can serve as evolutionary testbeds for new functions, and as scaffolds for engineering fibril‐based catalysts through sequence modification, repeat grafting, or directed evolution.

A third perspective arises from **mechanical function**. Amyloid fibrils exemplify hierarchical design: at the molecular scale, the crystalline β‐sheet cores of FapC reach GPa stiffness; at the mesoscale, single fibrils and mats fall in the MPa range; and at the ecological scale of hydrated biofilms, elasticity lies in the kPa regime. This hierarchy shows how molecular order translates into ecological resilience, enabling biofilms to withstand environmental fluctuations. In applied contexts, such scale‐aware mechanics could be harnessed to design extracellular scaffolds with tunable stiffness for biotechnology or materials science.

These molecular and mechanical insights also open up **ecological and medical perspectives**. FuBAs may have enabled bacteria to colonize extreme or fluctuating environments, where fibril stability ensured biofilm integrity under stress. Moreover, their ability to cross‐seed with host amyloids such as α‐Syn and Aβ hints at a deeper role in host–microbe co‐evolution, potentially influencing neurodegenerative disease trajectories. This duality presents both opportunities and risks: FuBAs could be therapeutic targets in microbiome‐linked amyloid diseases, but also serve as reproducible models for screening anti‐amyloid drugs, offering advantages over fragile human amyloid systems.

Finally, advancing the field will require **methodological integration**. Cryo‐EM provides atomic‐level detail for twisted fibrils, while SAXS offers complementary insights for untwisted assemblies. Molecular dynamics simulations capture fibril dynamics, and the growing role of machine learning and AI‐assisted modeling accelerates structure prediction and functional annotation. Laboratory evolution experiments, where the repeat number is experimentally altered and ecological outcomes monitored (e.g., robust biofilm improving fitness against physical or biological attack), could directly test the adaptive potential of modularity. Such integrative strategies promise to link molecular design with evolutionary trajectories and technological applications.

In conclusion, FuBAs demonstrate how imperfection at the sequence level yields precision at the structural and functional level. IRs provide the organizing principle that unites robustness, adaptability, and innovation. By bridging molecular evolution, microbial ecology, and biomaterials engineering, FuBAs stand as both biological exemplars and technological templates for designing multifunctional nanostructures.

## Conflict of Interest

The authors declare no conflict of interest.

## Supporting information



Supporting Information
