# Supplementary material for: The Importance of Being Imperfect: Structure and Function of Bacterial Amyloid
Source: Adv Sci (Weinh). 2025 Dec 5;13(4):e17090. doi: 10.1002/advs.202517090 (PMC12822426; doi:10.1002/advs.202517090)
Supplement: Supplementary file 1 — Supporting Information [file ADVS-13-e17090-s001.docx]

Supporting Information

The importance of being imperfect: Structure and function of bacterial amyloid

*Samuel Peña-Díaz*, Yanting Jiang, Zhefei Zhang, Anders Daugberg, Pedro Ferreira, Marcos López Hernández, Chandrika Mittal, Maria Joao Ramos, Jan Skov Pedersen, Morten Kam Dahl Dueholm, Cao Qin, Huabing Wang* and Daniel Otzen**

**Phylogenetic analysis**

HMM models for the Fap and Curli genes ^[1]^ were used as queries to search for homologs in the protein sequences of all species representative genomes in the Genome Taxonomy Database version 226. The search was performed twice using the hmmsearch command from HMMER version 3.4 (<http://hmmer.org/>); once with the --tblout flag (for detection of homologous genes) and once with the --domtblout flag (for detection of homologous domains in genes). The results from these searches were parsed with a custom python script to convert them into tidy csv files. A custom python script was then used to cluster all hits from the homology search which were located within 5000 bp of each other. Data exploration of these gene clusters was performed in separate Rmarkdown files for the Fap and Curli system using several tidyverse packages.^[2]^ An e-value cut-off for the gene homologs of 10^-5^ was set. If there was more than one HMM hit in a gene, only the one with the highest bit score was kept. The dataset was filtered to only include gene clusters which contained a hit for the imperfect repeat HMM (FapBC_repeat for Fap, Csg_AB for Curli), and at least one other Fap/Curli HMM. The Fap operons detected this way had a remarkably conserved synteny, which allowed us to assign genes detected by the FapBC_repeat HMM as either FapB or FapC based on their position in the operon. In the Curli operons this was only possible for a subset of the detected operons, due to a much higher variability in synteny. The genes from the filtered dataset were extracted and used to annotate the phylogenomic tree of all species representatives from GTDB v226 using the R package ggtree and ggtreeExtra.^[3]^ The imperfect repeat sequence logos were generated from the imperfect repeats detected in the extracted genes and were visualised using the R package ggseqlogo.^[4]^ All code necessary to reproduce the phylogenetic analysis can be found at <https://github.com/AOHD/amyloid_review/>.

**Computational Models**

Atomistic models of trimeric CsgA and FapC for MD simulations were constructed from cryo-EM structures (PDB codes: 8ENQ and 9U4U, respectively). Each amyloid was placed in a periodic rectangular water box with a minimum distance of 12 Å from any solute atom to the box boundary and solvated with the OPC3 water model. Protein residues were described with the ff19SB force field, while the four small-molecule substrates, ampicillin, nitrocefin, p-nitrophenyl acetate (pNPA), and p-nitrophenyl butyrate (pNPB), were parameterized using GAFF2 atom types using AmberTools24 suite. RESP partial charges for each ligand were derived from HF/6–31G(d) electrostatic potential calculations performed with Gaussian 16.

For each amyloid–ligand pair, we generated complexes using two approaches: (1) blind molecular docking with GNINA 1.3 ^[5]^ using an exhaustiveness parameter for the global search of 512 and the Vinardo scoring function, retaining the highest-scored pose; and (2) random ligand placement within the simulation box using the GROMACS 2025.3 insert-molecules tool. For each preparation method, six independent MD simulations were performed, yielding 12 independent trajectories per amyloid–ligand pair and 48 total simulations.

Each system underwent three sequential energy minimization stages using the steepest descent algorithm (maximum 50,000 steps). Two minimizations with decreasing harmonic position restraints on the amyloid backbone and ligand heavy atoms, followed by an unrestrained minimization. Minimized structures served as starting points for all subsequent MD simulations.

Each MD simulation comprised three stages: (i) NVT equilibration (2 ns, 1 fs time step) to heat the system to 310.15 K using the v-rescale thermostat with a harmonic restraint of 400 kJ mol⁻¹ nm⁻² on the amyloid backbone and ligand heavy atoms; (ii) NPT equilibration (5 ns, 1 fs time step) at 310.15 K and 1 bar using the C-rescale barostat with reduced restraints (100 kJ mol⁻¹ nm⁻²); and (iii) production MD (500 ns, 2 fs time step) in the NPT ensemble at 310.15 K and 1 bar without positional restraints.

All MD simulations were performed with GROMACS 2025.3 employing the Verlet cutoff scheme. Short-range electrostatic and Lennard-Jones interactions were truncated at 10 Å, with Lennard-Jones forces subject to potential-shift–Verlet smoothing and long-range dispersion corrections. All bonds involving hydrogen atoms were constrained using the LINCS algorithm, enabling a 2 fs time step during production runs. Per amyloid–ligand pair, we executed 12 production trajectories of 500 ns each, providing 6.0 µs of total sampling per system.

**SAXS analysis**

All-atom models of fibrils were obtained as described before.^[6]^ Briefly, AF ^[7]^ predictions of trimers were obtained, and the center monomer was used as a fibril precursor. Then, using the script S1 in pymol, the monomer was aligned to the Z-axis, replicated, and the copies were translated and rotated (if necessary) to a final quantity of monomers that exceeds the q-range resolution. Different angles and implementation of different disorders within the non-fibril domains (using S2) were evaluated using AUSAXS ^[8]^ against the data sets obtained previously [6].

*Script S1*

#howtouse: load script to pymol and run the formula as: build_and_align_fibril("monomer", "fibril", num_repeats=40, translation=14.0, twist_angle=-2.5); change “monomer” by the monomer name and the specific parameters.

from pymol import cmd import numpy as np import random

def compute_fibril_axis(num_repeats=40, translation=14.0, twist_angle_deg=-2.5): axis = np.array([0, 0, 1]) pos = np.zeros(3) R_total = np.eye(3)

for _ in range(num_repeats):
 angle_rad = np.deg2rad(twist_angle_deg)
 c, s = np.cos(angle_rad), np.sin(angle_rad)
 Rz = np.array([
 [c, -s, 0],
 [s, c, 0],
 [0, 0, 1]
 ])
 R_total = Rz @ R_total
 pos += R_total @ (axis * translation)

return pos / np.linalg.norm(pos)

def align_fibril_to_z_axis(object_name, num_repeats=40, translation=14.0, twist_angle_deg=-2.5): fibril_vec = compute_fibril_axis(num_repeats, translation, twist_angle_deg) print("Computed fibril vector:", fibril_vec)

target_vec = np.array([0, 0, 1])
v = np.cross(fibril_vec, target_vec)
s = np.linalg.norm(v)
c = np.dot(fibril_vec, target_vec)

if s < 1e-8:
 if c > 0:
 print("Fibril already aligned with Z-axis.")
 return
 else:
 print("Fibril is anti-aligned. Rotating 180 degrees.")
 perp = np.cross(fibril_vec, [1, 0, 0])
 if np.linalg.norm(perp) < 1e-8:
 perp = np.cross(fibril_vec, [0, 1, 0])
 perp = perp / np.linalg.norm(perp)
 cmd.rotate(perp.tolist(), 180, object_name)
 return

v /= s
K = np.array([
 [0, -v[2], v[1]],
 [v[2], 0, -v[0]],
 [-v[1], v[0], 0]
])
R = np.eye(3) + K + (K @ K) * ((1 - c) / (s ** 2))

matrix = [
 R[0, 0], R[0, 1], R[0, 2], 0.0,
 R[1, 0], R[1, 1], R[1, 2], 0.0,
 R[2, 0], R[2, 1], R[2, 2], 0.0,
 0.0, 0.0, 0.0, 1.0
]

cmd.transform_object(object_name, matrix)
cmd.origin(object_name)
cmd.center(object_name)
cmd.zoom(object_name)

def replicate_along_axis_spiral(object_name, num_repeats, offset, output_name, twist_angle=10): cmd.set("connect_mode", 0) axis = np.array([0, 0, 1]) names = [] chain_ids = "ABCDEFGHIJKLMNOPQRSTUVWXYZabcdefghijklmnopqrstuvwxyz0123456789"

R_total = np.eye(3)
pos = np.zeros(3)

for i in range(num_repeats):
 new_name = f"{object_name}_copy_{i}"
 cmd.create(new_name, object_name)

 translated_vector = R_total @ (axis * offset)
 pos += translated_vector
 cmd.translate(pos.tolist(), new_name)

 angle_deg = (i + 1) * twist_angle
 cmd.rotate([0, 0, 1], angle_deg, new_name)

 if i < len(chain_ids):
 cmd.alter(new_name, f"chain='{chain_ids[i]}SrS'")
 r, g, b = random.random(), random.random(), random.random()
 color_name = f"color_{i}"
 cmd.set_color(color_name, [r, g, b])
 cmd.color(color_name, new_name)

 Rz = np.array([
 [np.cos(np.deg2rad(twist_angle)), -np.sin(np.deg2rad(twist_angle)), 0],
 [np.sin(np.deg2rad(twist_angle)), np.cos(np.deg2rad(twist_angle)), 0],
 [0, 0, 1]
 ])
 R_total = Rz @ R_total

 names.append(new_name)

cmd.create(output_name, " + ".join(names))
for name in names:
 cmd.delete(name)

def build_and_align_fibril(monomer_name="monomer", fibril_name="fibril", num_repeats=40, translation=14.0, twist_angle=-2.5): print("⏳ Aligning monomer...") align_fibril_to_z_axis(monomer_name, num_repeats, translation, twist_angle)

print("🧱 Building fibril...")
replicate_along_axis_spiral(monomer_name, num_repeats, translation, fibril_name, twist_angle)

print("🎯 Centering and orienting...")
cmd.center(fibril_name)
cmd.zoom(fibril_name)
cmd.orient(fibril_name)

*Script S2*

from pymol import cmd

import random

# === USER PARAMETERS ===

pdb_file = "fibrilPAOnotwist.pdb" # Your input file

chains = ["A","B","C","D","E","F","G","H","I","J","K","L","M","N","O","P","Q","R","S","T","U","V","W","X","Y","Z","a","b","c","d","e","f","g","h","i"]

nterm_residues = 18 # Number of N-terminal residues to rotate

loop_regions = [(172,260)] # List of (start, end) loop residue indices

n_models = 5 # Number of randomized models

max_angle = 60 # Maximum rotation angle in degrees

min_cycles = 500 # Number of minimization cycles for each rotated part

# =========================

cmd.load(pdb_file, "prot")

def get_atom_coords_safe(selection):

"""

Return coordinates of the first atom in a selection.

Warn if multiple atoms are found.

"""

m = cmd.get_model(selection)

if len(m.atom) == 0:

raise ValueError(f"[ERROR] No atoms found for selection: {selection}")

if len(m.atom) > 1:

print(f"[WARN] Selection {selection} returned {len(m.atom)} atoms, using the first one.")

return list(m.atom[0].coord)

def random_unit_vector():

vec = [random.uniform(-1, 1) for _ in range(3)]

norm = sum(x**2 for x in vec) ** 0.5

return [x / norm for x in vec]

for model_id in range(n_models):

model_name = f"model{model_id}"

cmd.create(model_name, "prot")

for chain in chains:

# ---- N-terminal rotation ----

try:

pivot_sel = f"chain {chain} and resi {nterm_residues} and name C and not elem H"

pivot = get_atom_coords_safe(pivot_sel)

axis = random_unit_vector()

sel = f"({model_name} and chain {chain} and resi 1-{nterm_residues})"

angle = random.uniform(-max_angle, max_angle)

print(f"[INFO] Rotating chain {chain} N-term (1–{nterm_residues}) by {angle:.1f}° around random axis")

cmd.rotate(axis, angle, sel, origin=pivot, camera=0)

print(f"[INFO] Minimizing N-term region of chain {chain}")

cmd.minimize(selection=sel, cycles=min_cycles)

except Exception as e:

print(f"[WARN] Could not rotate N-term for chain {chain}: {e}")

# ---- Loop rotations ----

for (start, end) in loop_regions:

try:

pivot_sel = f"chain {chain} and resi {start} and name CA and not elem H"

pivot = get_atom_coords_safe(pivot_sel)

axis = random_unit_vector()

sel = f"({model_name} and chain {chain} and resi {start}-{end})"

angle = random.uniform(-max_angle, max_angle)

print(f"[INFO] Rotating chain {chain} loop ({start}–{end}) by {angle:.1f}° around random axis")

cmd.rotate(axis, angle, sel, origin=pivot, camera=0)

print(f"[INFO] Minimizing loop {start}-{end} of chain {chain}")

cmd.minimize(selection=sel, cycles=min_cycles)

except Exception as e:

print(f"[WARN] Could not rotate loop {start}-{end} in chain {chain}: {e}")

**References**

[1] a) M. S. Dueholm, D. Otzen, P. H. Nielsen, *PLoS One* **2013**, *8* (10), e76630, https://doi.org/10.1371/journal.pone.0076630; b) M. Dueholm, M. Albertsen, D. E. Otzen, P. H. Nielsen, *PLoS One* **2012**, *7*, e51274, https://doi.org/10.1371/journal.pone.0051274.

[2] H. A. Wickhman, A.; Bryan, J.; Chang, W.; D'Agostino, L.M.; François, R.; Grolemund, G.; Hayes, A.; Henry, L.; Hester, J.; Kuhn, M.; Pedersen, T.L.; Miller, E.; Bachem S. M.; Müller, K.; Ooms, J.; Robinson, D.; Seidel, D.P.; Spinu, V.; Takahashi, K.; Vaughan, D.; Wilke, C.; Woo, K.; Yutani, H. , *The Journal of Open Source Software* **2019**, *4(43), 1686*, https://doi.org/10.21105/joss.01686.

[3] a) S. Xu, L. Li, X. Luo, M. Chen, W. Tang, L. Zhan, Z. Dai, T. T. Lam, Y. Guan, G. Yu, *Imeta* **2022**, *1* (4), e56, https://doi.org/10.1002/imt2.56; b) S. Xu, Z. Dai, P. Guo, X. Fu, S. Liu, L. Zhou, W. Tang, T. Feng, M. Chen, L. Zhan, T. Wu, E. Hu, Y. Jiang, X. Bo, G. Yu, *Mol Biol Evol* **2021**, *38* (9), 4039, https://doi.org/10.1093/molbev/msab166.

[4] O. Wagih, *Bioinformatics* **2017**, *33* (22), 3645, https://doi.org/10.1093/bioinformatics/btx469.

[5] A. T. McNutt, Y. Li, R. Meli, R. Aggarwal, D. R. Koes, *J Cheminform* **2025**, *17* (1), 28, https://doi.org/10.1186/s13321-025-00973-x.

[6] Y. Jiang, S. Peña-Díaz, Z. Zhang, A. O. H. Daugberg, M. López Hernández, J. Nielsen, Q. Huang, S. Qin, M. K. D. Dueholm, M. Dong, J. S. Pedersen, Q. Cao, D. E. Otzen, H. Wang, *Adv Mater* **2025**, e2505503, https://doi.org/10.1002/adma.202505503.

[7] J. Abramson, J. Adler, J. Dunger, R. Evans, T. Green, A. Pritzel, O. Ronneberger, L. Willmore, A. J. Ballard, J. Bambrick, S. W. Bodenstein, D. A. Evans, C. C. Hung, M. O'Neill, D. Reiman, K. Tunyasuvunakool, Z. Wu, A. Zemgulyte, E. Arvaniti, C. Beattie, O. Bertolli, A. Bridgland, A. Cherepanov, M. Congreve, A. I. Cowen-Rivers, A. Cowie, M. Figurnov, F. B. Fuchs, H. Gladman, R. Jain, Y. A. Khan, C. M. R. Low, K. Perlin, A. Potapenko, P. Savy, S. Singh, A. Stecula, A. Thillaisundaram, C. Tong, S. Yakneen, E. D. Zhong, M. Zielinski, A. Zidek, V. Bapst, P. Kohli, M. Jaderberg, D. Hassabis, J. M. Jumper, *Nature* **2024**, *630* (8016), 493, https://doi.org/10.1038/s41586-024-07487-w.

[8] K. Lytje, J. S. Pedersen, *Journal of Applied Crystallography* **2025**, *58* (4), 1332, https://doi.org/10.1107/S160057672500562X.
